# Evapolectrics: Direct Harvesting of Electricity from Evaporation Using Thermoelectrics

**DOI:** 10.1021/acsnano.5c10693

**Published:** 2025-07-11

**Authors:** Jing Cao, Jinfeng Dong, Jing Wu, Ady Suwardi

**Affiliations:** † Institute of Materials Research and Engineering, Agency for Science, Technology and Research, 2 Fusionopolis Way, 138634 Singapore; ‡ Department of Materials Science and Engineering, National University of Singapore, 117574 Singapore, Singapore; § School of Materials Science and Engineering, 54761 Nanyang Technological University, 50 Nanyang Ave, 639798 Singapore; ∥ School of Electronic Science & Engineering, 12579 Southeast University, Nanjing 211189, China; ⊥ Department of Electronic Engineering, and Shun Hing Institute of Advanced Engineering, The Chinese University of Hong Kong, Shatin, New Territories, Hong Kong SAR 999077, China

**Keywords:** evapolectrics, hydrovoltaics, thermoelectrics, water evaporation, electricity harvesting, water–energy nexus

## Abstract

Evaporation, a ubiquitous process driving Earth’s water–energy cycle, has been largely untapped for energy harvesting. Here, we introduce “evapolectrics,” a scalable strategy that directly converts evaporation enthalpy into electricity via thermoelectric generators (TEGs). By leveraging porous graphite coatings and optimizing wind speeds (2.8 m/s) and wet-bulb depression, a robust temperature gradient (Δ*T*) over 6 °C can be maintained across TEGs. This translates to a power density of 4.2 W/m^2^, which exceeds other ambient energy harvesting technologies, such as triboelectric and hydrovoltaics. We also demonstrate the evapolectrics’ ability to sustain a continuous power output of 2.72 mW over 30 min and scalability via a 7 × 7 device array. Unlike intermittent sources like solar or wind, evaporation’s perennial nature offers reliable ambient energy harvesting. With global evaporation rates suggesting harvestable energy of ∼10^5^ TJ/year, evapotetics present a transformative approach to power self-sustaining devices, augmented by advances in thermoelectric materials.

## Graphical abstract

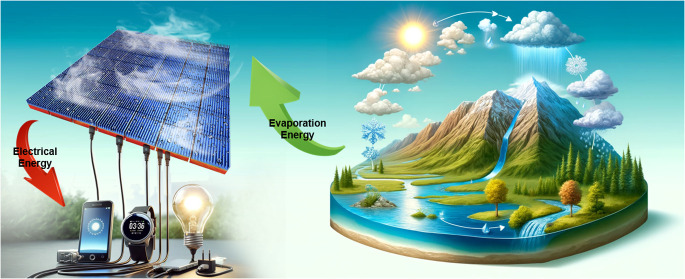

Water and sunlight are vital for life, forming a water–energy nexus that sustains organisms and shapes the climate and biology. Renewable technologies like solar cells, hydropower, and wind turbines largely rely on this cycle.^1^ Evaporation, a dominant energy transfer process, consumes up to 50% of solar energy absorbed by Earth’s surface, yet the attention to utilize water evaporation has so far been mainly focused on cooling technologies such as evaporative coolers. In terms of energy harvesting, efforts to directly convert evaporation into electricity have only emerged in recent years, largely focusing on two major areas, solar-driven evaporation and hydrovoltaics.^2,3^ Owing to its potential application in desalination and clean water harvesting, solar-driven evaporation has rapidly gained attention in recent years.^4–6^ Likewise, hydrovoltaics, which harvests energy from water gradients, droplets, and humidity, has been touted for its ability to scavenge power from the ambient environment.^7–10^ However, compared to other ambient energy harvesting technologies such as indoor photovoltaics, piezoelectric/triboelectric nanogenerators, and radiative cooling, hydrovoltaics tend to produce low power densities in the range of 10s of mW/m^2^.^10–17^ This can be ascribed to hydrovoltaics’ inherently indirect conversion process, through mechanical or chemical processes. Importantly, such a level of power density is insufficient for small electronics.^2^

Fundamentally, water evaporation takes away thermal energy, as defined by the enthalpy of vaporization, which also forms the basis of evaporative cooling. An overlooked class of materials for converting this cooling directly into electricity is thermoelectrics. Thermoelectrics provide direct conversion of heat (Δ*T*) into electricity and, conversely, electricity into cooling.^18–21^ In recent years, a series of breakthroughs have drastically improved thermoelectric materials’ figure of merits (*zTs*). *zTs* around 3 have been regularly reported in various compounds.^22^ Closer to room temperature, emerging materials such as Mg_3_Sb_2–*x*_Bi_*x*_ have also been widely studied and reported.^23^ Such advancements in materials performance are fundamentally driven by ingenious strategies of decoupling phonon and electronic transports, resulting in low thermal conductivity and high electrical mobilities.^24^ On the other hand, progress in interface materials and mechanical properties has also played a crucial role in the design of high-performance thermoelectric devices. As a result, ultraefficient power generation and cooling have been consistently predicted and reported.^25,26^ Nevertheless, for power generation, the traditional paradigm in thermoelectrics has always been about converting waste heat (temperatures higher than ambient) into electricity. Unfortunately, waste heat is limited compared with sunlight, wind, or hydropower. Thus, the market size of thermoelectrics is much smaller than that of photovoltaics or the wind industry.^27^ So far, thermoelectrics have been underexplored for direct harvesting of electricity from water evaporation. The first direct harvesting of electricity from evaporation was only reported recently in 2024 by Cheng et al.^28^

In this study, we harness the enthalpy of water evaporation to generate electricity directly using thermoelectric generators (TEGs), with only a few reports in the literature. Unlike conventional approaches, we actively establish and sustain a temperature gradient (Δ*T*) through water evaporation (Figure 1A). Under ideal conditions, as defined by a given air temperature (dry-bulb temperature, *T*_dry‑bulb_ from herein) and relative humidity (*RH*), the cold-side heat sink can theoretically reach the wet-bulb temperature *T*_wet‑bulb_, the minimum achievable via evaporation. The resulting *T*_dry‑bulb_ – *T*_wet‑bulb_ difference, termed wet-bulb depression, drives a Δ*T* between the cold-side (*T*_2_) and hot-side (*T*_1_) heat sinks. Notably, directional heat flow from evaporation reduces *T*_1_ below ambient air temperature (*T*_air_). To demonstrate scalability and practical utility, we fabricated a 7 × 7 device array (total area of 0.49 m^2^), capable of charging a smartphone and smartwatches (Supporting Information Video S3). We term this approach “evapolectrics” for brevity.

**Figure 1:**
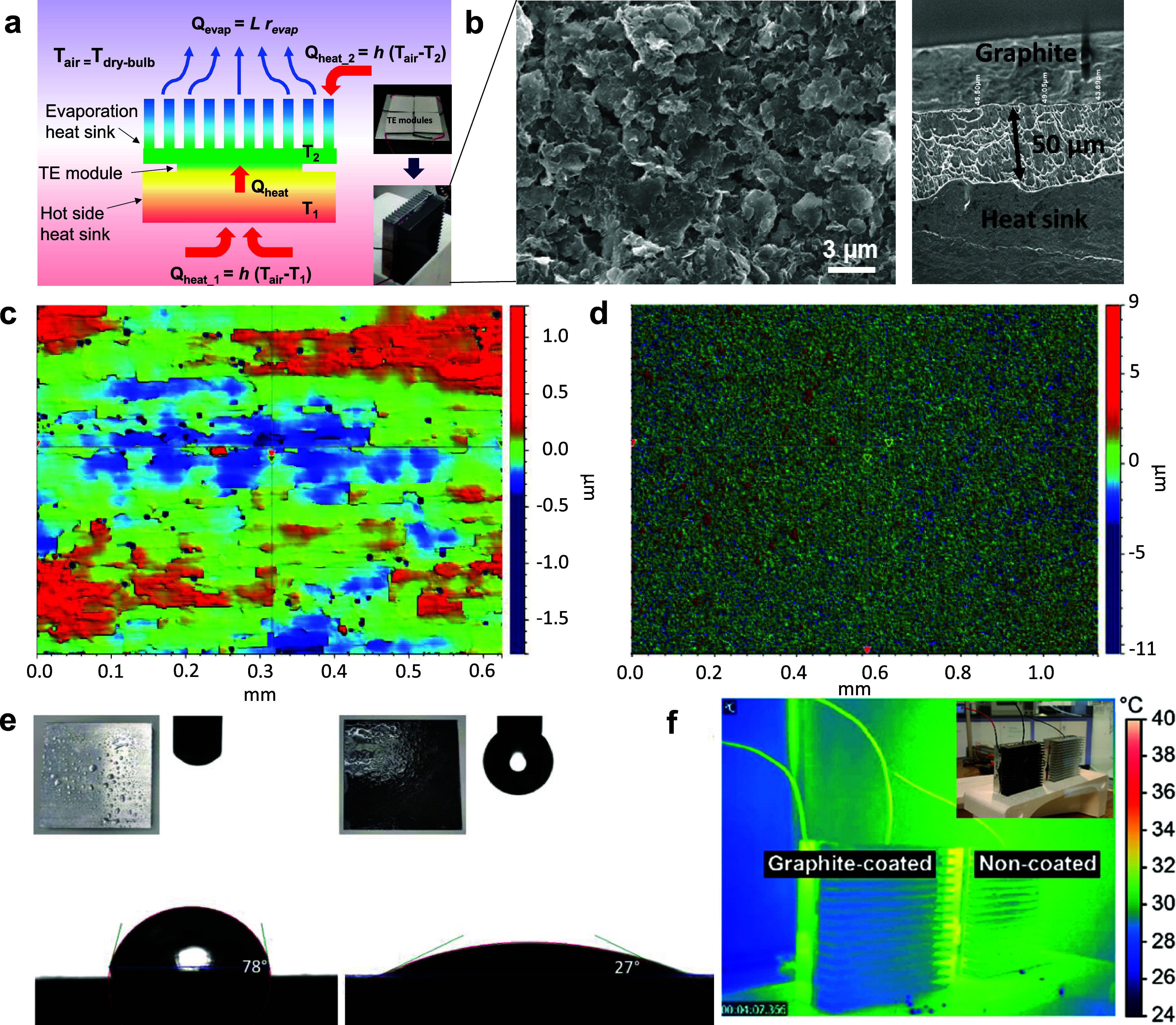
Illustration and device configuration of evapolectrics. (a) Schematic drawing showing the structure of an evapolectric device. The inset shows four TEG modules connected in series, sandwiched between two heat sinks. The heat sink on the cold side serves to increase the surface area to enhance evaporation, while the heat sink on the hot side serves as a heat exchanger with the surrounding air to maintain a higher Δ*T*. Red and blue arrows denote the quasi-1-dimensional heat transport direction. *Q*_evap_, *L*, *r*_evap_, *Q*_heat*_*1_, *Q*_heat*_*2_, and *h* represent the evaporation heat flux, latent heat of vaporization, evaporation rate, heat flux through heat sinks 1 and 2, and heat exchange coefficients, respectively. (b) Porous nature of the graphite coating under scanning electron microscopy, with 50 μm thickness. (c) Surface topology scan using a while light interferometer of pristine aluminum surface compared to (d) porous graphite-coated surface. (e) Wetting angle of water on aluminum surface vs graphite surface, showing reduced contact angle for porous graphite coating. (f) Thermal camera comparison between graphite-coated and noncoated aluminum heat sinks.

Efficient and sustained evaporation requires enhanced water retention on the evaporating heat sink surface. To this end, we applied a 50 μm thick porous graphite coating (Figure 1B). Surface topology analysis reveals that pristine aluminum exhibits an average roughness of <0.5 μm (Figure 1C, measured via white-light interferometry), whereas the graphite coating increases roughness to >4.0 μm (Figure 1D). Such an increase in roughness is intended to improve wettability (i.e., reduce effective wetting angle, θ_eff_), in accordance with the Wenzel angle (cos θ_eff_ = *r* cos θ), where *r* and θ_eff_ represent roughness factor and intrinsic contact angle. To experimentally verify this, the wetting contact angle analysis was done on the porous graphite surface. In its wet state, it exhibits a significantly lower contact angle (greater hydrophilicity) than aluminum (Figure 1E). Such observation is consistent with the expected decrease in wetting angle with increasing roughness. In addition to hydrophilicity, the porosity of the graphite coating also promotes a higher effective surface area (*A*_eff_) for evaporation via the simplified wetting model *A*_eff_ ∝ *r* cos θ_eff_. In this case, with an effective *r* of 1.5 (based on a roughness of 4 μm for a 50 μm thick coating) and θ_eff_ of 27°, the *A*_eff_ is around 1.34, or 34% higher than that of a flat surface. Consequently, the graphite-coated device demonstrates superior evaporation performance, evidenced by a lower, more uniform temperature profile compared to that of the uncoated device (Figure 1F).

## Results and Discussion

### Temperature Gradient, Wind Speed, and Porous Graphite Coating

To illustrate the effect of wind speed on temperature gradient, a computational fluid dynamics (CFD) simulation was used to demonstrate the cooling effect brought about by water evaporation, as demonstrated by the temperature profile and water mass fraction in Figure 2A (Supporting Information Videos S1 and S2). Interestingly, both the temperature and water mass fraction follow similar profiles, further supporting our premise that the cooling was brought about by water evaporation. As shown in the illustration in Figure S5, when an optimal amount of water is introduced to a porous material (analogous to watering the soil), capillary water condition will be achieved. As far as evaporation is concerned, capillary water represents the optimal condition where a substantial portion of the capillary water is available for evaporation, combined with high surface area due to the porosity and roughness relative to aluminum (Figures S2 and S3). Such an observation is analogous to agriculture, where capillary water represents the available amount of water to be absorbed by plants. To further exemplify the importance of capillary water, Figure S6 shows the effect of changing the amount of water introduced on the open-circuit voltage. According to the heat sink geometry used in our experiment, a porous film of 50 μm thickness can hold approximately 2.0 mL of water. It is evident that increasing the volume of water below the threshold of the capillary has a direct consequence on increasing the performance and output of the device. However, beyond 2.0 mL, the performance plateaus and even decreases slightly. This can be associated with gravitational water, at which point the excessive presence of water hinders the heat transfer through the heat sink to the TEG module, thereby lowering the output.

**Figure 2:**
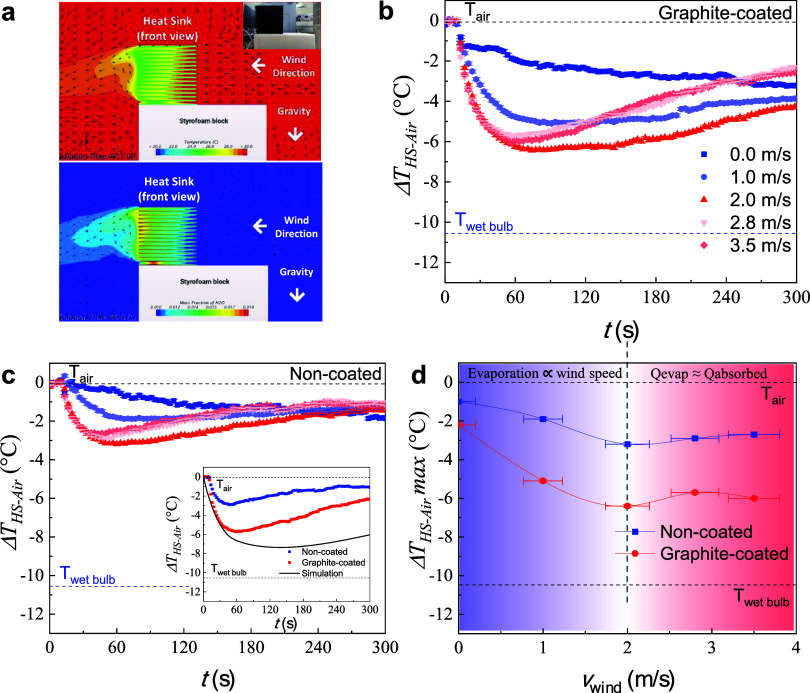
Temperature profiles of the water-evaporation side of the heat sink. (a) Thermal camera comparison between graphite-coated and noncoated aluminum heat sinks. The graphite-coated heat sink shows more uniform temperature distribution (left) compared to noncoated ones. In addition, it also shows a lower temperature and longer retention of temperature (right). (b, c) Temperature profile of graphite-coated vs noncoated heat sinks as a function of time and wind speed. Wind speed serves to increase evaporation and hence Δ*T*_HS‑Air_. However, further increasing wind speed beyond 2.0 m/s also results in increased heat absorption from the surrounding air, resulting in a plateau of maximum Δ*T*_HS‑Air_. Increasing wind speed also increases the evaporation/drying rate, resulting in faster decay from maximum Δ*T*_HS‑Air_ (poorer temperature retention). The inset of (c) shows the comparison of temperature profiles between graphite-coated, noncoated, and CFD-simulated values in Supporting Information Video S1. (d) Maximum Δ*T*_HS‑Air_ vs wind speed for both graphite-coated and noncoated heat sinks, showing a higher magnitude of Δ*T* for the graphite-coated heat sink at all wind speeds, as well as a plateau of maximum Δ*T*_HS‑Air_ beyond 2.0 m/s wind speed.

In addition, air movement or wind speed affects the evaporation. To examine the effect of wind speed and to emulate a real-life scenario, a gentle and directional breeze ranging from 1.0 to 3.5 m/s was introduced in our evapolectrics. These values are close to the global average wind speed of 3.28 m/s at sea level.^29^ Essentially, for outdoor applications, this represents the almost ever-present gentle breeze, which is ubiquitous enough to be harvested. Furthermore, the minimum wind speed (cut-in speed) to drive even a mini wind turbine is >3 m/s, which means the range of optimal wind speed in this work does not compete with wind power (i.e., cannot be harvested by a wind turbine).^30^ In addition, for indoor applications such as wearable electronics, the average walking speed of a healthy adult is 1.42 m/s.^31^ When combined with swing movements of arms, airflow speed around the wearable device can realistically average around 2–3 m/s.^32^ Therefore, the range of wind speed investigated in this work is carefully selected to represent realistic application conditions.

The effect of wind speed on water evaporation rate can be described by the following general equation, modified from Lewis and Whitman^33^

1 $$r_{evap}=A\left[\frac{Q_{\mathrm{in}}}{\left[L+C_{p}\left(T_{s}-T_{w}\right)\right]}+\rho \left(25+19\nu_{\mathrm{wind}}\right)\left(\chi_{S}-\chi_{A}\right)\right]$$

where *r*_evap_, *A*, *Q*_in_, *L*, *c*_p_, *T*_s_, *T*_w_, ρ, *ν*_wind_, χ_S_, and χ_A_ represent the evaporation rate (kg/s), surface area (m^2^), external input power (W/m^2^), latent heat of vaporization (J/kg), constant pressure specific heat (J/kgK), surface water temperature (K), bulk water temperature (K), density (kg/m^3^), wind speed (m/s), and saturated and actual vapor content in air (kg/kg), respectively. For natural evaporation, the first part of the equation is zero as there is no external energy input, *Q*_in_ = 0 (this is distinct from solar-driven thermal evaporation, where *Q*_in_ is the absorbed thermal energy).^34^ The second part of the equation suggests a linear dependence of evaporation rate on wind speed. However, a more nuanced view of χ_S_ – χ_A_ suggests temperature and relative humidity dependence, which are nontrivial functions of wind speed.

Figure 2B shows the evolution of cooling temperature (Δ*T*_HS‑Air_) of the heat sink with respect to air temperature (*T*_dry‑bulb_) as a function of time and wind speed for a graphite-coated heat sink. To enable consistent comparison, the *T*_dry‑bulb_ and relative humidity (*RH*) were kept at 31 °C and 40%, respectively. As expected, an increase in wind speed leads to a higher magnitude of Δ*T*_HS‑Air_ due to enhanced evaporation. Maximum Δ*T*_HS‑Air_ over 6 °C was achieved at 2.0 m/s wind speed and beyond. Interestingly, further increasing wind speed results in poorer retention of Δ*T*_HS‑Air_ (faster decay), which can be ascribed to a faster evaporation/drying rate of the heat sink and increased heat transfer from the surrounding air. The dashed blue line denotes *T*_wet‑bulb_, which represents the theoretical lowest temperature achievable via evaporation at a particular *T*_dry‑bulb_ and *RH*. The maximum Δ*T*_HS‑Air_ achieved in the experiment does not reach *T*_wet‑bulb_, which can be ascribed to heat transfer from the hot-side heat sink (Figure 1B). Intuitively, lowering the thermal conductivity of TEG may push the magnitude of Δ*T*_HS‑Air_ closer to *T*_wet‑bulb_. In addition, strategies such as improving thermal insulation within the spaces between the two heat sinks and geometric optimization of legs within TEG (i.e., hourglass shape instead of cuboid) can also bring the Δ*T*_HS‑Air_ closer to *T*_wet‑bulb_.^35^ Furthermore, the cooling profile of a noncoated heat sink is provided in Figure 2C for comparison. Although the trend of Δ*T*_HS‑Air_ with respect to wind speed remains similar, the magnitude of Δ*T*_HS‑Air_ is much lower compared to that of a graphite-coated heat sink, as shown in the inset of Figure 2C for 2.8 m/s wind speed and Figure 2D at various wind speeds. This can be ascribed to increased surface area for evaporation due to the graphite coating, consistent with eq 1. It is noteworthy that at wind speed >2.0 m/s, heat absorption from surrounding air also increases, compensating for the heat loss by evaporation, preventing further increase in magnitude of Δ*T*_HS‑Air_.

### Electrical Output Characteristics

The Δ*T*_HS‑Air_ described in Figure 2B–D represents how much the cold-side heat sink gets cooled by evaporation. However, a quasi-1D heat flow from the hot-side heat sink to the cold-side heat sink (Figure 1B) means that the temperature in the hot-side heat sink is also lower than the surrounding air temperature (*T*_dry‑bulb_). As such, the actual Δ*T* across the thermoelectric generators (TEGs) is different from Δ*T*_HS‑Air_, as are its trend and profile as a function of wind speed. Across a thermoelectric device, the most accurate indicator of the actual Δ*T* is the open-circuit voltage (*V*_OC_) generated. Figure S7 shows the comparison of *V*_OC_ temporal profiles between noncoated vs graphite-coated devices at different wind speeds (Supporting Information Video S5). For consistent comparison, *T*_dry‑bulb_ was kept at 31 °C and *RH* at 40%. The time-dependent profile as a function of wind speed is shown in Figure S8A,B for noncoated and graphite-coated devices, respectively. The graphite-coated device shows higher *V*_OC_ compared to noncoated counterpart, which attests to the importance of ensuring high evaporation rate to create high Δ*T* (Figures S7 and S8C,D). Interestingly, for both graphite-coated and noncoated devices, the maximum *V*_OC_ were observed at a wind speed of 2.8 m/s (Figure S8C,D). This contrasts with the trend for Δ*T*_HS‑Air_ in Figure 2, which shows an optimal wind speed of 2.0 m/s. Such an observation is expected because higher wind speed increases the heat intake in the hot-side heat sink (Figure 1B), which helps to maintain a higher Δ*T* across the TEGs.

In every experiment, only 2 mL (2g) of water is sprayed on the heat sink (127 g). Although water has a higher heat capacity compared to aluminum, the substantial difference in weight renders the effect of water temperature insignificant due to drastically lower thermal mass. To verify this, a control experiment was done at *RH* 99% to ensure negligible cooling effect from evaporation, as shown in Figure 3A. Evidently, while the sign of *V*_OC_ is opposite between 20 and 40 °C water, they are largely transient in nature, with a magnitude much lower compared to *V*_OC_ in Figure S7. Furthermore, at *RH* 40% and *T*_dry‑bulb_ 31 °C, no significant difference in *V*_OC_ can be observed using water at different temperatures (Figure 3B), consistent with the lower thermal mass of water compared to a heat sink.

**Figure 3:**
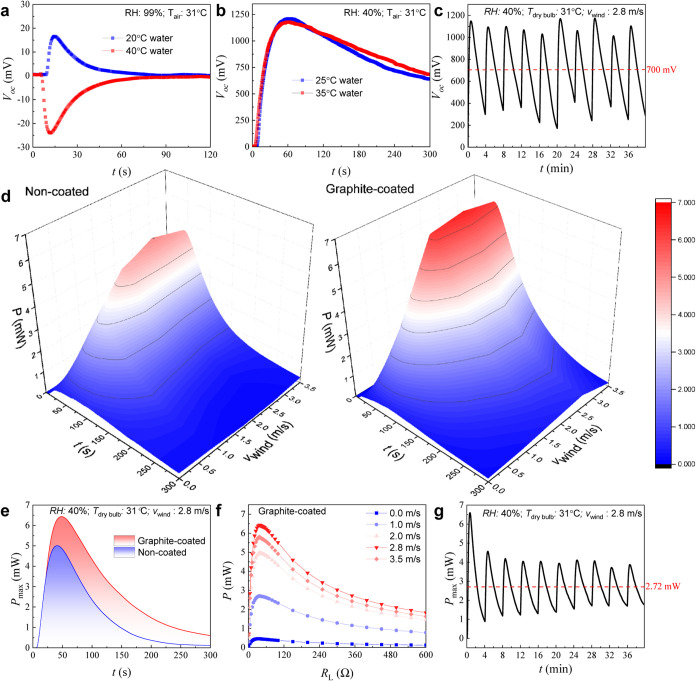
Electrical output characteristics of evapolectrics. (a) To rule out the effect of water temperature on heat sink temperature, *V*_OC_ profiles of water temperature above and below room temperature are compared at *RH* 99% (negligible evaporation). Evidently, *V*_OC_ from water temperature is transient, with a much lower magnitude compared to *V*_OC_ in Figure 2A. (b) *V*_OC_ profiles of different water temperatures at the same *T*_dry‑bulb_ and *RH* as in Figure 2A, confirming the negligible effect of water temperature. (c) Long-term *V*_OC_ profile with periodic water introduction (1.6 mL every 4 min) showing consistent, robust, and sustainable output over a prolonged period. An average *V*_OC_ of 700 mV was maintained over 40 min. (d) Comparison of output power profiles between noncoated vs graphite-coated devices at different wind speeds at *T*_dry‑bulb_ 31 °C and *RH* 40%. Consistent with the Δ*T* data in Figure 2, the output power for graphite-coated is higher than that for noncoated ones. In terms of the wind speed effect, the highest power is notably observed at 2.8 m/s, which is slightly higher than the optimal wind speed for Δ*T* in Figure 2. (e) Comparison of output power at impedance matching condition for noncoated vs graphite-coated devices, which shows higher power (30% higher) and sustained over longer period. This is consistent with a longer retention of Δ*T*_HS‑Air_ in the graphite-coated device observed in Figure 2A. (f) Power vs load resistance (*R*_L_) curves for the graphite-coated device at various wind speeds. The impedance matching condition is achieved at *R*_L_ 40 Ω, and the highest power at a wind speed of 2.8 m/s, consistent with Figure 2A. (g) Long-term output power at impedance-matched condition for graphite-coated device showing sustainable power over a prolonged period, with an average of 2.72 mW over 40 min. Notably, the profile in panel (g) differs from that in panel (d). Lower power can be observed after the initial cycle of water introduction, which is associated with the Peltier effect due to current flow in a closed-circuit condition.

To demonstrate repeatability and continuity in evapolectrics, Figure 3C shows the long-term *V*_OC_ profile (1.6 mL of water is introduced every 4 min interval) over 40 min, with an average *V*_OC_ of 700 mV. To further evaluate the useful power generated from evapolectrics, closed-circuit experiments with impedance-matched conditions were conducted, as shown in Figure 3D. Expectedly, the power generated for a graphite-coated device is markedly higher than noncoated one. For comparison, Figure 3E shows a higher (30%) output power from the graphite-coated device compared to that from a noncoated one. Interestingly, the power for the graphite-coated device can also be sustained over a longer period, consistent with a longer retention of Δ*T*_HS‑Air_ in Figure 3A. The external load resistance required for achieving impedance matching is determined from power vs load resistance curves in Figure 3F for the graphite-coated device and Figure S9A for the noncoated device, which shows an optimum *R*_L_ of 40 Ω to achieve impedance matching (Supporting Information Video S6). It is noteworthy that just like *V*_OC_, the maximum power, *P*_max_, for all wind speeds of graphite-coated devices is higher than that of noncoated devices (Figure S9B). Lastly, Figure 3G shows the long-term output power under impedance-matched conditions for the graphite-coated device. An average power of 2.72 mW can be sustained over 40 min. Notably, the profile in Figure 3G differs from that in Figure 3C because lower power can be observed after the initial cycle of water introduction. This is associated with the Peltier effect due to current flow in the closed-circuit condition, which serves to compensate for the actual Δ*T* across the TEGs.

### Psychrometrics and Power Generation

A psychrometric chart provides a holistic and intuitive platform for analyzing otherwise complex interdependencies between evaporation, *T*_dry‑bulb_, and *RH*. As the figure of merit of evapolectrics is to achieve high Δ*T* via evaporation, it is versatile to plot a psychrometric chart in three axes: *T*_dry‑bulb_, *RH*, and *T*_wet‑bulb_, as shown in Figure 4A. For a given *T*_dry‑bulb_ (vertical line), its intersection with *RH* (curved lines) can be used to determine the *T*_wet‑bulb_, which is the thermodynamic lowest achievable temperature via evaporation. The region in green represents the range of the experimental parameters in this work. It is noteworthy that the range of *T* and *RH* covered in this work falls within the thermal comfort range as defined by ISO 7730:2005 (PMV) index, which represents realistic atmospheric conditions in our daily lives. Figure 4B shows the maximum power, *P*_max_ (at wind speed 2.8 m/s) as a function of all combinations of *RH* and *T*_dry‑bulb_ in Figure 4A (corresponding *V*_OC_ shown in Figure S10). The time-dependent profiles at various *RH* and *T*_dry‑bulb_ are shown in Figures S11–S25, respectively. The highest *V*_OC_ is achieved at the lowest range of *RH* and the highest *T*_dry‑bulb_, which combines to provide a high evaporation rate and a high Δ*T* across TEGs. Notably, the effect of varying *RH* (red dotted lines) on *V*_OC_ is more significant compared to the effect of changing *T*_dry‑bulb_ (blue dotted lines). This verifies the significant role of evaporation in creating a robust Δ*T*. In addition to evaporation, from a thermoelectric materials point of view, the Δ*T* can be further enhanced by designing better materials. However, it is important to add that the thermoelectric materials design criteria for efficiency may not necessarily revolve around the traditional figure of merit (*zT*) due to their finite heat flux operation.

**Figure 4:**
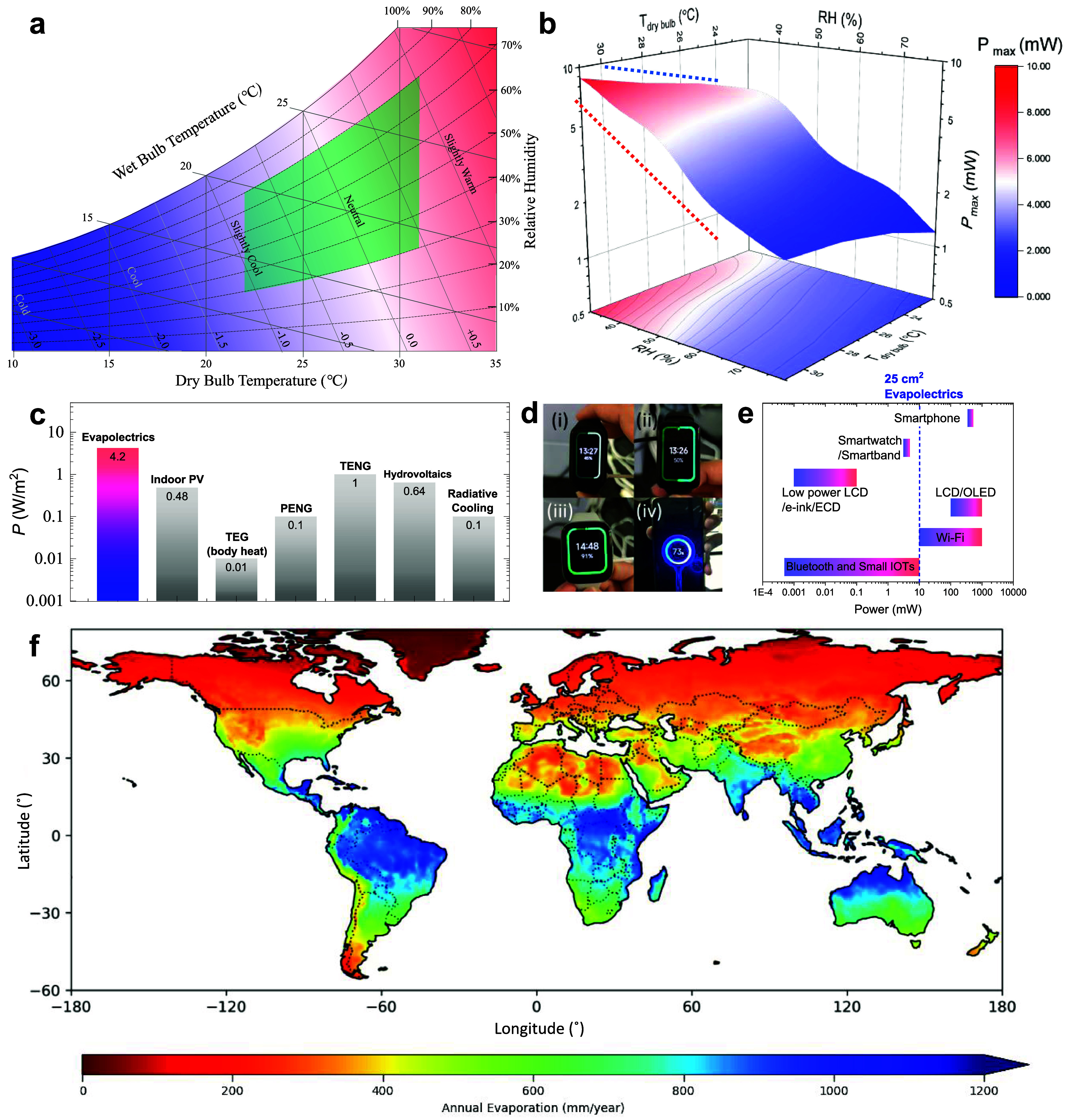
Psychrometric chart showing psychrometrics and power density of evapolectrics under different atmospheric conditions. (a) Psychrometric chart showing *T*_dry‑bulb_ (temperature measured by thermometer), *T*_wet‑bulb_ (lowest achievable temperature due to evaporation), and relative humidity (%). The region in green represents the range of experimental parameters in this work. It is noteworthy that the range of T and RH covered in this work falls within the thermal comfort range as defined by the ISO 7730:2005 (PMV) index, which represents realistic atmospheric conditions in our daily lives. (b) Maximum power, *P*_max_ (at wind speed 2.8 m/s), as a function of all combinations of RH and *T*_dry‑bulb_ in panel (a). The highest *V*_OC_ is achieved at the lowest range of RH and the highest *T*_dry‑bulb_, which combines to provide a high evaporation rate and a high Δ*T* across TEGs. Notably, the effect of varying RH (red dotted lines) on *V*_OC_ is more significant compared to the effect of changing *T*_dry‑bulb_ (blue dotted lines). This verifies the central role of evaporation in creating robust Δ*T*. (c) Comparison of power density achieved by evapolectrics with other ambient energy harvesting technologies: indoor photovoltaics (PV),^13^ body-heat TEG,^10^ PENG (piezoelectric nanogenerators),^10^ TENG (triboelectric nanogenerators),^36,37^ hydrovoltaics,^11^ and radiative cooling.^38^ In addition to high power density and ubiquity, evapolectrics do not suffer from the intermittency problem. It is worth noting that the active materials used in evapolectrics are TEGs. However, the power density generated from evapolectrics is much higher compared to body-heat TEG due to the higher heat flux caused by evaporation compared to body-heat flux. (d) Demonstration of evapolectrics power used to charge (i) fitness tracker, (ii) smartband, (iii) smartwatch, and (iv) smartphone. To achieve the charging effect, a 7 × 7 array of evapolectric devices was electrically connected in series, and thermally in parallel, with careful thermal insulation (Supporting Information Video S3). (e) Range of continuous power requirements for various small electronics devices to achieve battery-less utilization. Blue dashed line represents the power generated by a 25 cm^2^ evapolectric device (palm size), suggesting its far-reaching potential for providing continuous power for myriads of low-power devices. (f) Global distribution of mean annual evaporation, the data set was obtained from CRU-NCEP^39^ and CERES,^40^ with the plotting method based on ref 41.

Figure 4C shows the comparison of power density achieved by electrodynamics with other ambient energy harvesting technologies. In addition to high power density and ubiquity, evapolectrics do not suffer from the intermittency problem. It is worth noting that the active materials used in evapolectrics are TEGs. However, the power density generated from evapolectrics is much higher compared to body-heat TEG due to the substantially higher heat flux caused by evaporation compared to body-heat flux. As far as body-heat-powered wearables are concerned, evapolectrics open the exciting possibility of augmenting the heat flux from body-heat and heat flux from evaporation, further increasing the power density. Furthermore, to demonstrate the scalability and practicality of evapolectrics, a 7 × 7 array of devices was designed and used to charge (i) a fitness tracker, (ii) a smartband, (iii) a smartwatch, and (iv) a smartphone in Figure 4D. Finally, Figure 4E shows the range of continuous power requirements for various small electronics devices to achieve battery-less utilization. Blue dashed line represents the power generated by the 25 cm^2^ evaptic device (palm size), suggesting its far-reaching potential for providing continuous power for myriads of low-power devices. Finally, although the evapolectric device and output shown in this work are in terms of mWs, it is important to note that evaporation is both ubiquitous and perennial. As shown in Figure 4F, the average global evaporation rate from March 2000 to December 2016 can reach up to 5 mm/day on land and >5 mm/day on water bodies. Such an evaporation rate translates to around 10^8^ Tera Joules (TJ) of evaporation enthalpy energy per year. Assuming a modest TEG conversion efficiency of 0.1%, this translates to 10^5^ TJ per year of harvestable energy or 20 GW of energy. This is equivalent to powering approximately 20 million homes or around 4 million electric vehicles simultaneously.

## Conclusions

Due to the dissipative nature of heat, evaporation energy has so far eluded any meaningful utilization or storage. For instance, although the vast majority of Earth’s surface is covered with water bodies, the cooling brought about by evaporation is quickly brought to equilibrium via thermal exchange with the surrounding environment. This makes the evaporation energy “unseen” to the community by and large. Our work on evapolectrics uncovers the virtually limitless potential of energy utilization/storage from natural evaporation. Notably, the 4.2 W/m^2^ power density of evapolectrics is 2 orders of magnitude higher than the current record for hydrovoltaics. In addition, owing to the noncompeting sources, evapolectrics have the potential to augment other ambient energy harvesting technologies such as body heat, triboelectric, indoor photovoltaics, and radiative cooling. Not to mention, novel phenomenon such as photomolecular effect may provide promising direction for evaporation energy harvesting.^34^ Overall, these technologies will greatly expand the possibilities of self-powered small electronics devices relying solely on ambient energy. Finally, with the ever-rapid progress in thermoelectric materials and devices, the realizable power density of evaptical materials will undoubtedly continue to increase in the years to come.

## Methods

### Evapolectric Device

Each evaptic device in this work consists of 4 thermoelectric generator (TEG) modules. TEG modules (model TES1–24125) with 40 mm × 40 mm lateral size and 4 mm thickness were obtained from Everredtronics Ltd. The 4 TEGs are electrically connected in series via solder. It is important to ensure that the polarity of the TEG modules is aligned with each other (facing the same way) to prevent compensation of the generated voltage. It is also crucial to ensure good thermal contact to enable heat flow from the hot side through the TEGs to the cold side of the heat sink with minimal temperature drop across the interface. For this purpose, Silicone thermal grease obtained from RS Pro (thermal conductivity of 5.0 Wm^–1^ K^–1^) was used to attach the TEG modules, which were attached to 100 mm × 100 mm aluminum heat sinks aligned perpendicularly to each other. The perpendicular orientation was done with a double purpose: for ease of arraying and for selectively increasing the heat exchange coefficient on the evaporation side to enable a faster rate of evaporation, while preventing bypass airflow on the hotter side to preserve the temperature gradient. For the scaling-up experiment involving a 7 × 7 array of devices, thermally conductive double-sided tape (Xiamen Naikos New Materials, Co. Ltd.), with <0.5 mm thickness and a thermal conductivity of 1.2 W/(m·K), was used instead of thermal grease. This was done to ensure adequate mechanical strength when the array of heat sinks is assembled. Another perpendicularly oriented aluminum heat sink was used to sandwich the TEG modules to complete the evapolectric device. For a graphite-coated heat sink, GRAPHITE spray from Cramolin was sprayed evenly on the surface of the heat sink. The thickness of the graphite coating ranges from 40 to 50 μm. The primary purpose of the spray is to ensure water retention and uniform spreading of water by leveraging the porosity of the coating.

### Relative Humidity and Temperature Setup

For controlling the experimental atmospheric condition, all experiments were carried out in an enclosed acrylic environment (1200 mm × 800 mm × 800 mm). To enable precise relative humidity control, both a dehumidifier (Xiaomi smart dehumidifier 22L) and a humidifier (Xiaomi Mijia humidifier 2) were used. For air temperature control, both a heat gun and an air cooler were used. For accurate reading of relative humidity and temperature, a sensor from AcuRite 01080 Pro was used after humidity calibration in a glovebox. For thermal insulation during the experiment, a polystyrene block was used to place the evapolectric device. For measuring the wind speed, a vane probe anemometer from RS Pro (193–8696) was used. This is negligible compared to the temperature gradient brought about by evaporation. For the experimental device temperature, an FLIR AX5 thermal camera was used to study the time evolution of temperature profiles of the heat sink. The temperature data was extracted from the average over the area of the heat sink temperature with autogain correction applied.

### Array of Evapolectrics and Charging Experiments

To demonstrate scalability, an array of 7 × 7 evaptical samples was prepared. For minimizing thermal loss, thin metal wires were used to hold the array of devices together, which are electrically connected in series. The entire setup was then sprayed uniformly with graphite spray on the evaporation side (cold side). To enable smartphone/smartwatch charging, water (at room temperature) was sprayed on the graphite-coated side (Supporting Information Video S3). The relative humidity and temperature during the experiment were set to be close to 40% and 31 °C, respectively. In total, voltages as high as 40–50 V were generated. To convert the stochastic output voltage to 5 V required for charging a smartphone and smartwatch, a step-up/step-down converter from Linear Technology, demo circuit 527A, model LT3433EFE, was used. The converter takes in any input voltage from 4 to 60 V and converts it into a 5 V output, with a maximum current of 400 mA.

### Electrical Measurements

To measure the open-circuit voltage (*V*_OC_) and output power (*P*), the evapolectric device was connected in series to a precision programmable resistance decade PRS-300 from IET Laboratories, Inc. PRS-300 was set in the four-probe mode, where the voltage connections were made to a Keithley 6430 subfemtoamp sourcemeter. The LabVIEW program was used to control both PRS-300 and Keithley 6430. For *V*_OC_ measurement, the resistance of PRS-300 was set to a maximum (20 MΩ) to ensure that no current was flowing in the circuit. For output power vs load resistance measurement, the PRS-300 resistance was swept from 1 to 600 Ω. For comparison, the typical impedance matching for an evapolectric device (consisting of 4 TEG modules) is around 40 Ω. Supporting Information Videos S5 and S6 illustrate the *V*_OC_ and *P* data collection, respectively. For the collection of *P*_max_ versus time data, the resistance at PRS-300 was set at 40 Ω, with voltage data collection done as a function of time. For long-term open-circuit voltage and output power collection, the PRS-300 resistance was set at 20 MΩ and 40 Ω, respectively, while a 1.6 mL water spray was introduced to the graphite-coated heat sink every 4 min intervals (except the initial spray of 2 mL).

### Computational Fluid Dynamics (CFD) Simulation

To simulate the transient profile of temperature and water content on the heat sink, CFD based on turbulence modeling (realizable K-Epsilon) was employed. The heat transfer model accounts for both conduction and convection while ignoring the radiative portion due to the low temperature gradient involved in the study. For the fluid flow, a multicomponent gas model was utilized: dry air and water vapor, with the fluid film model assumed on the surface of the evaporating heat sink. modeling: When modeling evaporation from multicomponent liquid films, the vapor pressure of each individual component and therefore its evaporation rate are dependent on the concentration of the different components in the mixture. In addition, gravity was also accounted for in the simulation. The experimental parameters used in the simulation are listed in Table S1.

### Effect of Ambient Lighting

In addition, an RS Pro IM720 light intensity meter was used to rule out the potential light-induced heating effect in the graphite-coated side. An intensity of around 170 lx was detected from ambient light, which translates to approximately 100 μK heating assuming 100% absorption. To rule out the effect of light intensity on the evapolectrics performance, a comparison was drawn with the experiment performed under dark conditions (<10 lx) and ambient indoor light (170 lx), as shown in Figure S30, with no substantial difference in performance observed between these conditions.

## Supplementary Material















## Data Availability

The data that support the findings of this study are available from the corresponding author on reasonable request.

## Notes

Heat flow equation, evapolectrics efficiency, surface characterization, wetting angle characterization, experimental setups, device setups, assumptions in literature comparison, and modeling parameters (PDF)
Computational fluid dynamics (CFD) simulation showing the time evolution of surrounding air temperature and front face heat sink temperature as the water evaporates. Wind direction and gravity orientation are indicated by respective arrows (MP4)
Computational fluid dynamics (CFD) simulation showing the time evolution of the mass fraction of water in the surrounding air and at the front face heat sink as the water evaporates. Wind direction and gravity orientation are indicated by respective arrows (MP4)
7 × 7 array of evapolectrics sprinkled with water showing enough power generated from evaporation to charge s smartphone (MP4)
Thermal imaging video showing the side-by-side comparison of the time evolution of temperature between noncoated and graphite-coated heat sinks (MP4)
Real-time LabVIEW video showing the data acquisition of VOC (open-circuit voltage) after water is introduced onto the heat sink surface. Video is fast-forwarded after 40 s (MP4)
Real-time LabVIEW video showing the output power vs load resistance after water is introduced onto the heat sink surface (MP4)
