# Supplementary material for: Evapolectrics: Direct Harvesting of Electricity from Evaporation Using Thermoelectrics
Source: ACS Nano. 2025 Jul 11;19(28):26249–58. doi: 10.1021/acsnano.5c10693 (PMC12291591; doi:10.1021/acsnano.5c10693)
Supplement: Supplementary file 1 [file nn5c10693_si_001.pdf]

Supplementary Information for

Evapolectrics: Direct Harvesting of Electricity from Evaporation Using Thermoelectrics

Jing Cao[1,2*], Jinfeng Dong[3], Jing Wu[4], Ady Suwardi[5*]

[1]Institute of Materials Research and Engineering, Agency for Science, Technology and Research; 2 Fusionopolis Way, 138634, Singapore.

[2]Department of Materials Science and Engineering, National University of Singapore 117574, Singapore, Singapore.

[3]School of Materials Science and Engineering, Nanyang Technological University; 50 Nanyang Ave, 639798, Singapore.

[4]School of Electronic Science & Engineering, Southeast University; Nanjing, 211189, China.

[5]Department of Electronic Engineering, and Shun Hing Institute of Advanced Engineering, The Chinese University of Hong Kong, Sha Tin, New Territories, Hong Kong SAR, 999077, Hong Kong.

*Corresponding author. Email: caoj@nus.edu.sg (J.C.); asuwardi@ee.cuhk.edu.hk (A.S.)

Heat flow equation

Neglecting heat loss through the thickness direction of the evapolectric, the heat transfer through the TEG modules can be treated as quasi 1 dimensional. The fundamental driving force to create ΔT across TEG in this case is evaporation, which takes away heat:

$$Q_{evap} = L\, r_{evap} \qquad (S1)$$

where $Q_{evap}, L,$ and r_{evap} represents evaporation heat flux (W, negative sign), latent heat of vaporization (J/Kg), and evaporation rate (Kg/s), respectively.

The evaporation from the heat sink will leave behind temperature depression (negative ΔT), resulting in the cold side heat sink with temperature $T_2 < T_{air}$. However, as $T_2 < T_{air}$, naturally the system will try to reach thermal equilibrium with the surrounding air:

$$Q_{heat\_2} = h(T_{air} - T_2) \qquad (S2)$$

where $Q_{heat_2}$and h represents heat gain from the environment (W, positive sign), and heat exchange coefficient with the environment (W/(m^2.K)). It is noteworthy that under ideal condition where there is no other heat exchange, the equilibrium between equation S1 and S2 will lead to a $T_{air} - T_2$ defined as wet-bulb depression, where T_{air} is the dry-bulb temperature, and T_2 is the wet-bulb temperature. For instance, under lower relative humidity (RH), r_{evap} will be higher (as defined by equation (1) in the main text, leading to a lower T_2 (wet-bulb temperature). Vice-versa is true.

In actual experiment, since the thermal conductivity of TEG modules are non-zero, quasi 1 dimensional heat will transfer from hot-side heat sink to cold side heat sink through the TEG modules:

$$Q_{heat} = \frac{\kappa A_{TEG}(T_1 - T_2)}{t} \qquad (S3)$$

where κ, A_{TEG}, and t represents effective thermal conductivity of TEG modules (W/(m.K)), effective cross sectional area of TEG (m^2), and thickness of the TEG (m). Intuitively from equation S3, one can deduce that lower thermal conductivity of TEG modules or thicker module will be helpful to maintain high ΔT (T_1-T_2) through the TEG. Lastly, it is worth adding that due to heat flow from hot side to cold side heat sink, T_1 is actually lower than T_{air}. Hence, there exist heat absorption at hot side heat sink defined as:

$$Q_{heat\_1} = h(T_{air} - T_1) \qquad (S4)$$

It is noteworthy that the presence of wind serves to increase the heat exchange coefficient (h), leading to lower $T_{air} - T_1$.

Evapolectrics Energy Conversion Efficiency ($\eta_{evapolectrics}$)

The device dimension in this work is 10 cm × 10 cm, giving a total effective evaporation area of 0.01 m^2. At moderate ambient conditions (31 °C, 40 % RH, 2.8 m/s wind). A stable thermoelectric power of 6.5 mW is established. If we operate the device continuously for 1 hour (3600 s), the total electrical energy output E_{out} is:

$$E_{\text{out}} = \int_0^{\Delta t} P\,dt \approx (6.5\ \text{mW}) \times (3600s) = 23.4\,J \qquad (S5)$$

From experimental values at aforementioned conditions, the evapolectric setup can achieve natural evaporation rate of about 0.7 kg/m^2h, which is about 7.0 g of water per hour. The latent heat of vaporization of water (L_{water}) at room temperature is approximately 2438 J g^{-1}.[1] Thus, the thermal energy spent to evaporate 2.58g of water is:

$$Q_{\text{in}} = \Delta m \times L_{water} = 7.0\ g \times 2438\ \text{Jg}^{-1} = 17066\,J \qquad (S6)$$

Therefore, the corresponding efficiency η can be calculated:

$$\eta_{evapolectrics} = \frac{E_{\text{out}}}{Q_{\text{in}}} \approx 0.14\% \qquad (S7)$$

The value suggests that approximately 0.1% of latent heat from water evaporation is converted to electricity under the stated operating conditions. It aligns with the performance of hydrovoltaic devices that harvest energy from natural evaporation processes.[2-7] Although it seems low compared to other energy harvesting systems, it is worth noting that our passive evaporation-driven hydrogel-TEG generator requires neither external energy input (e.g. sunlight, heat source, etc.) nor complex material and device configurations.

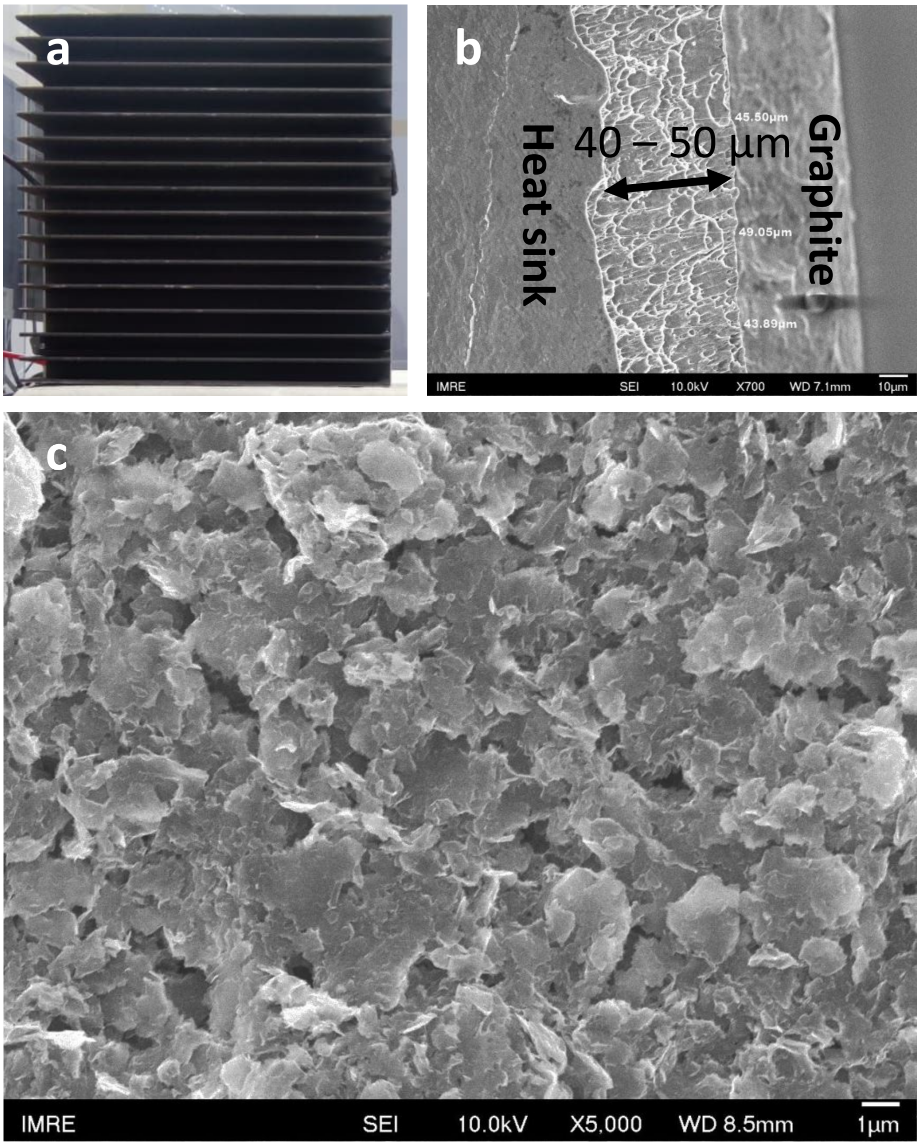

Fig. S1. Photo of graphite-coated evapolectric device and the microscopy of the graphite. **(a)** Front face of the graphite-coated heat sink **(b)** Thickness of the graphite coating under SEM. **(c)** Porous nature of the graphite coating.

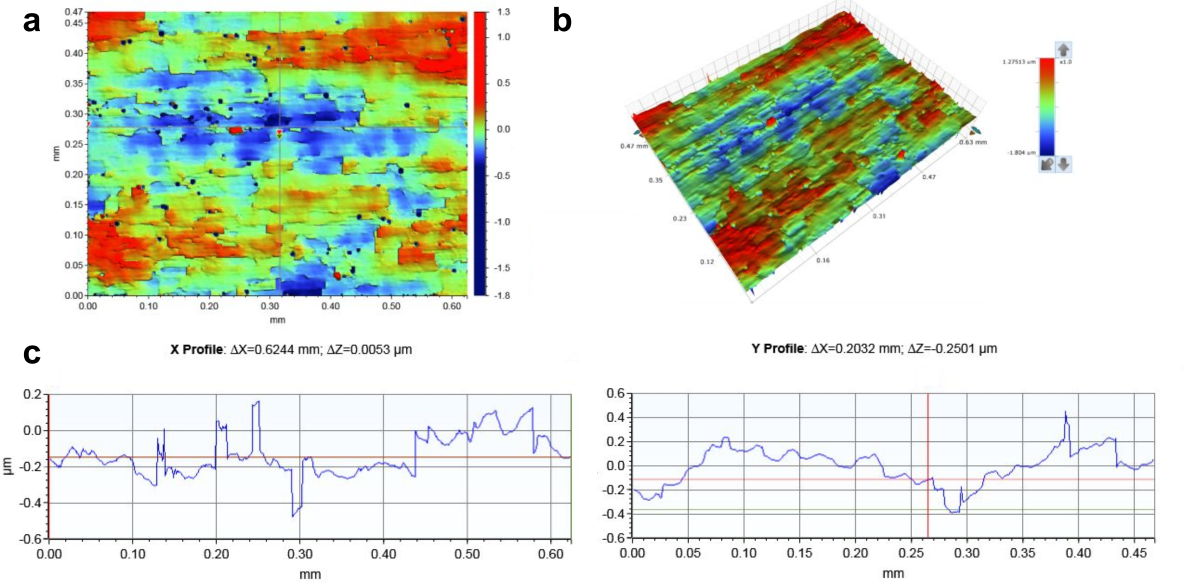

Fig. S2. (a) Surface topology scan using While Light Interferometer of pristine aluminium surface (b) roughness profile showing average roughness of < 0.5 µm. (c) Line scan across vertical and horizontal direction, showing sub-micron variation in roughness profile.

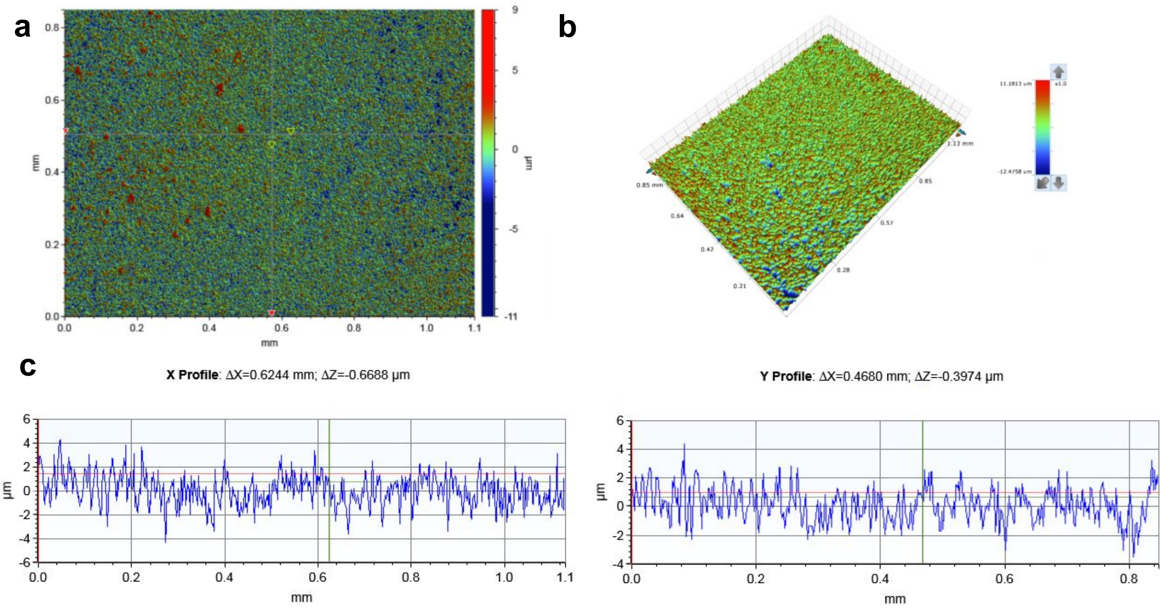

Fig. S3. (a) Surface topology scan using While Light Interferometer of graphite coating (b) roughness profile showing average roughness of > 1 µm. (c) Line scan across vertical and horizontal direction, showing sub-micron variation in roughness profile.

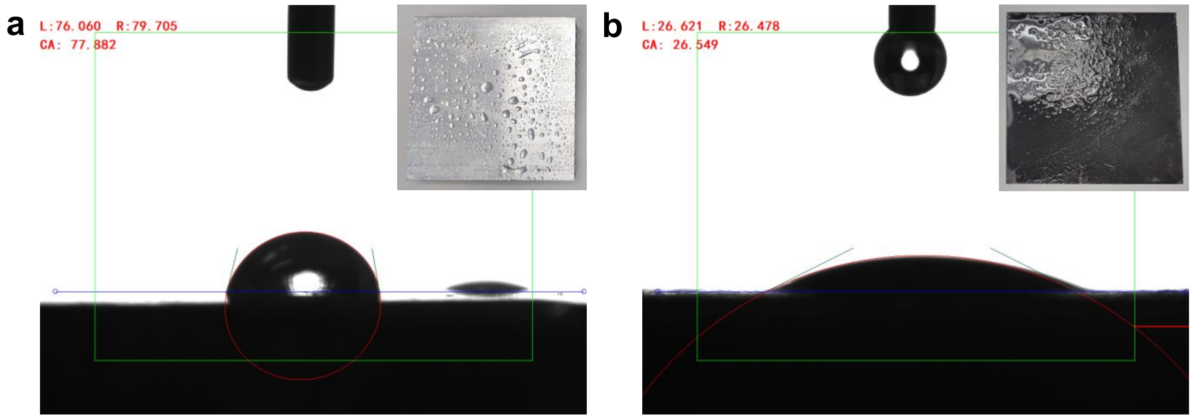

Fig. S4. Wetting angle of water on aluminum surface vs graphite surface, showing hydrophobic and hydrophilic nature, respectively.

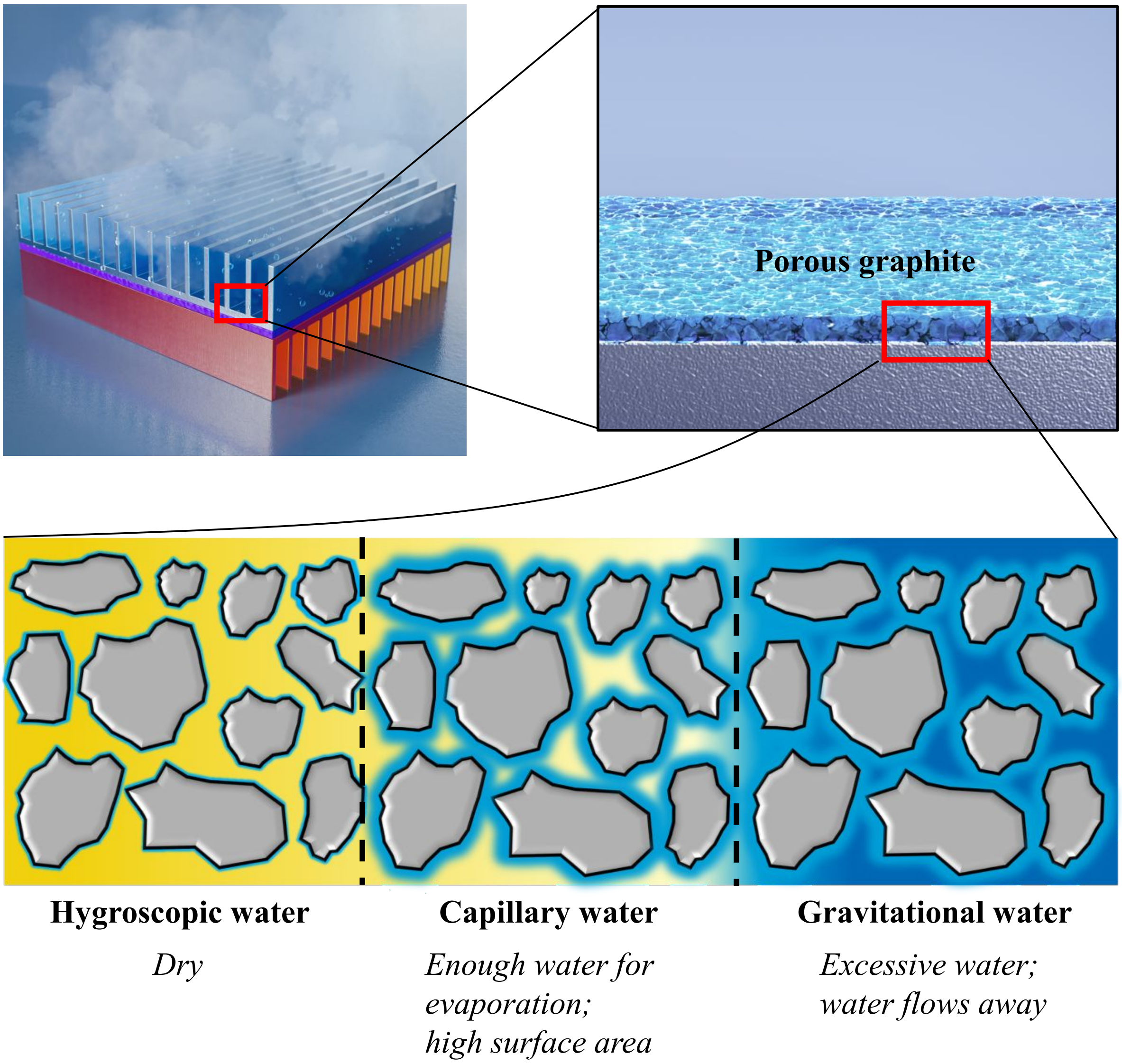

Fig. S5. Illustration showing the role of porous graphite coating in increasing water evaporation and water retention. When sufficient volume of water is present (capillary water), the evaporation can simultaneously be sustained for longer period due to higher water retention, with higher evaporation rate due to increased surface area.

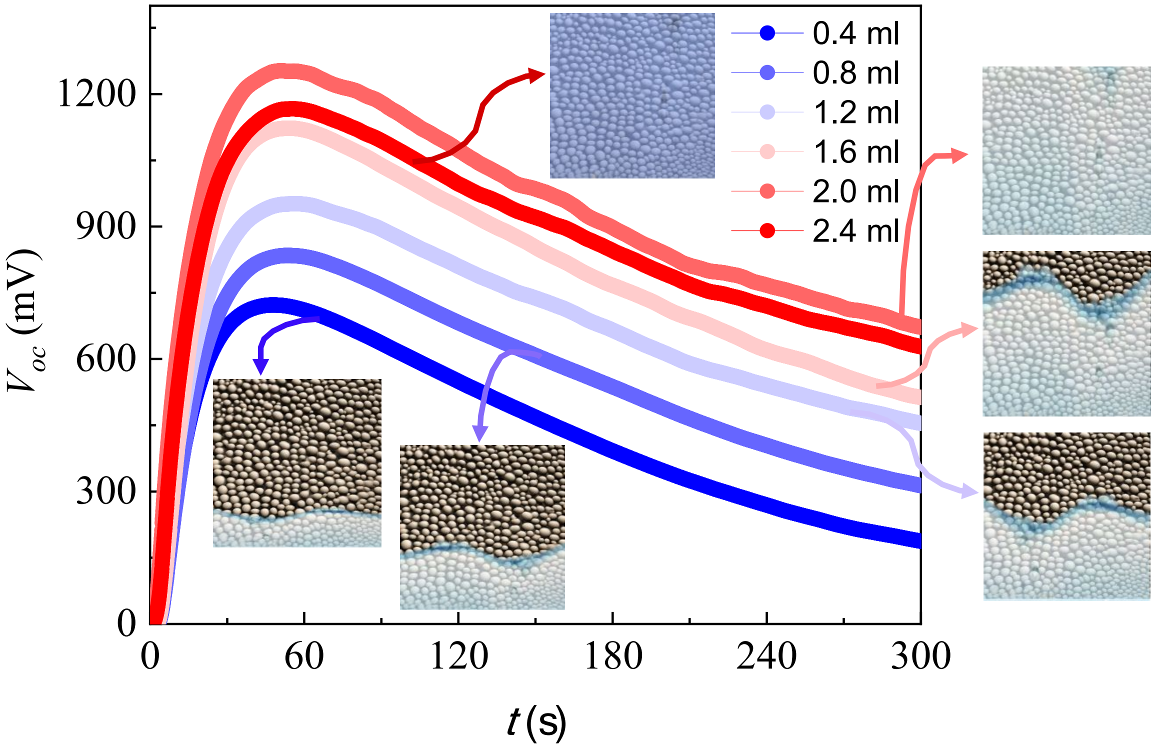

Fig. S6. Effect of water volume on the open circuit voltage profile of evapolectrics. Illustration represents the extent of capillary water in the porous graphite. All experiments were conducted at RH 40% and 31°C.

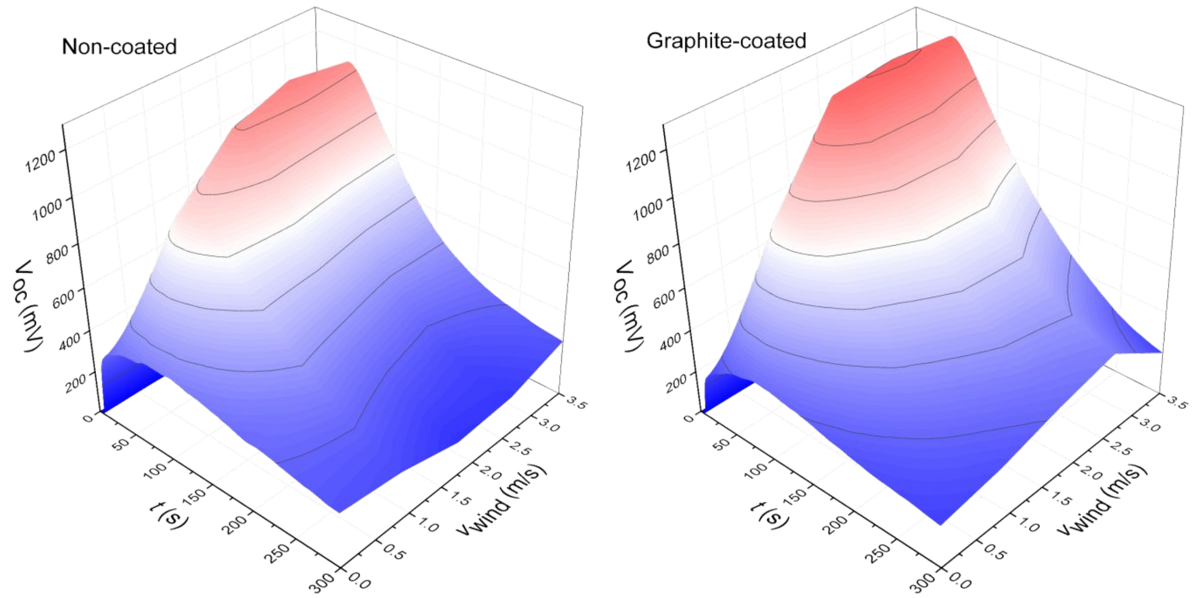

Fig. S7. Comparison of open-circuit voltage (V_{OC}) profiles between non-coated vs graphite-coated devices at different wind speed at $T_{dry\text{-}bulb}$ 31°C and RH 40%. Consistent with ΔT data in Fig.2, the V_{OC} for graphite-coated is substantially higher than non-coated ones. In terms of wind speed effect, the highest VOC is notably observed at 2.8 m/s, which is slightly higher than the optimal wind speed for ΔT in Fig.2.

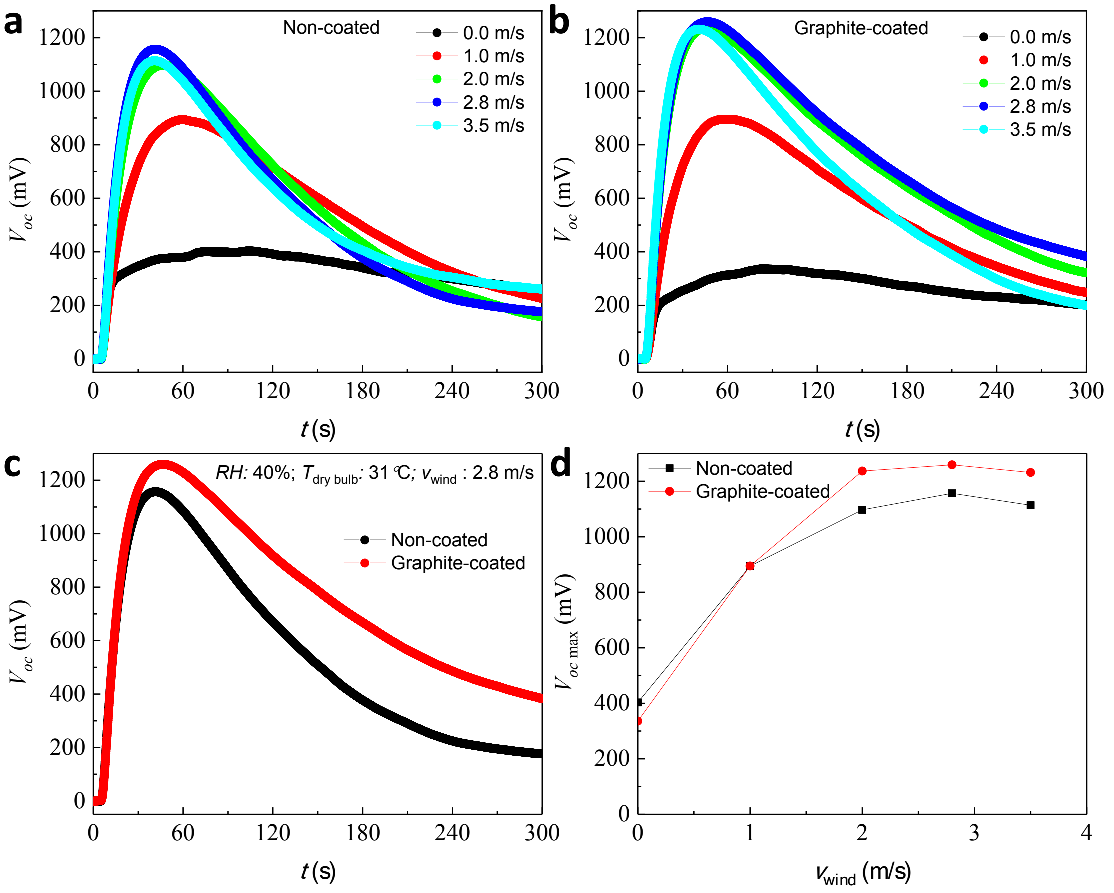

Fig. S8. Open-circuit voltage as a function of various wind-speed and time. (a) Non-coated evapolectrics. (b) graphite-coated evapolectrics. (c) Comparison of V_{OC} for non-coated vs graphite-coated evapolectrics at 2.8 m/s wind speed. (d) Maximum V_{OC} at various wind speeds for both non-coated and graphite-coated evapolectrics.

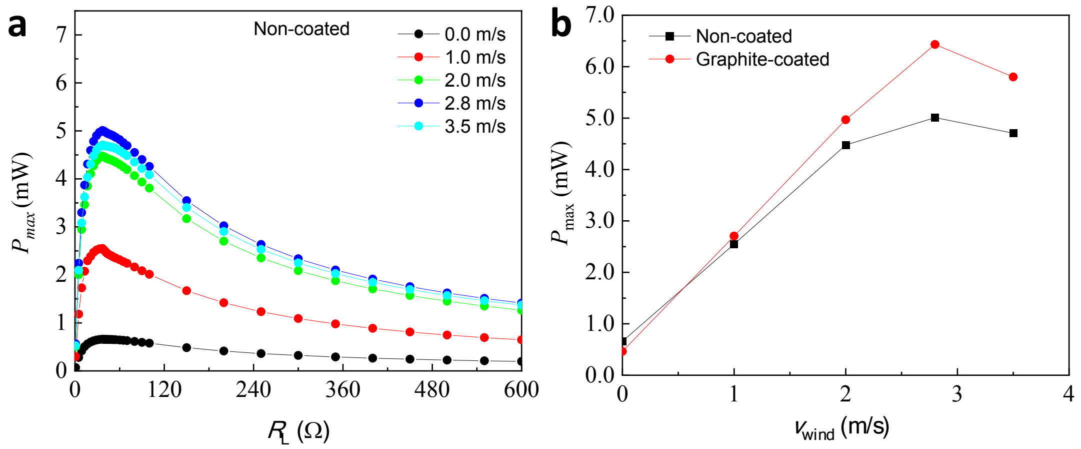

Fig. S9. Power output at impedance matching condition (P_{max}) for non-coated evapolectrics at various wind speeds. (a) as a function of load resistance. **(b)** P_{max} as the function of wind speeds.

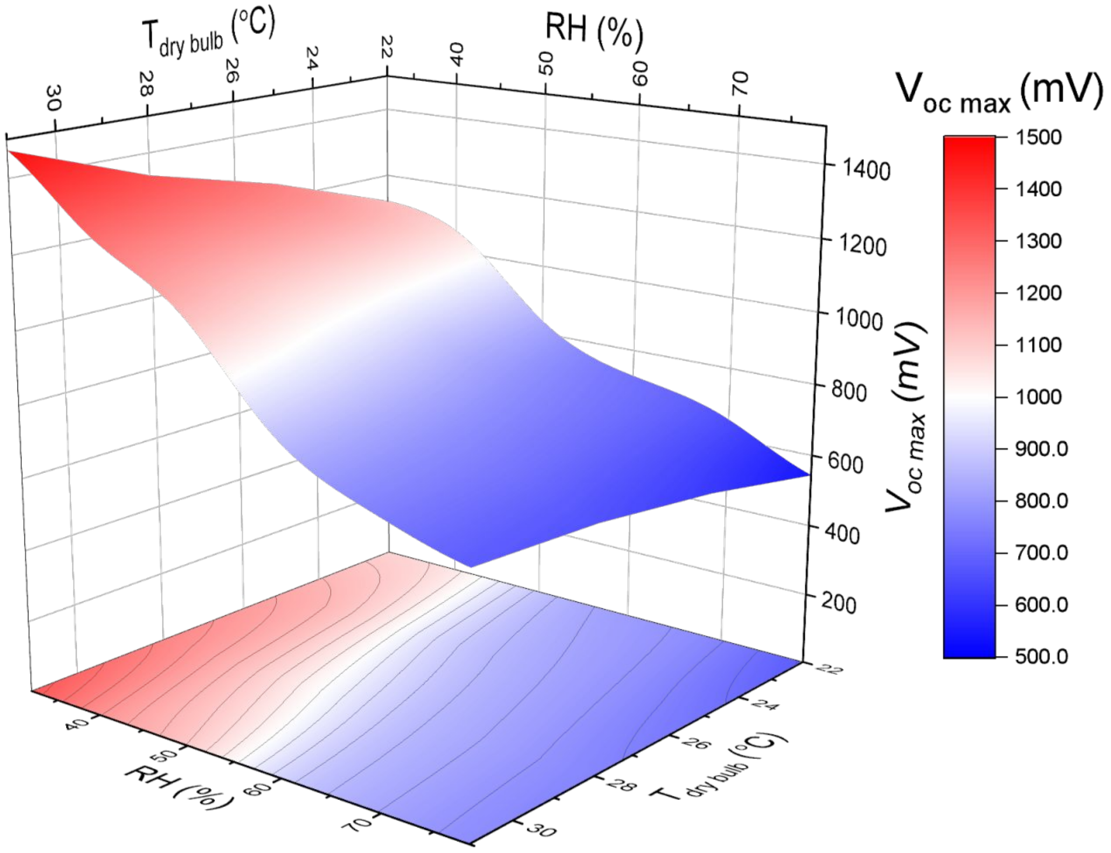

Fig. S10. Maximum V_{OC} (at wind speed 2.8 m/s) as a function of all combinations of RH and $T_{dry\text{-}bulb}$ in Fig. 4A. Highest V_{OC} is achieved at the lowest range of RH and highest $T_{dry\text{-}bulb}$, which combines to provide high evaporation rate and high ΔT across TEGs. Notably, the effect of varying RH (red dotted lines) on V_{OC} is more significant compared to the effect of changing $T_{dry\text{-}bulb}$ (blue dotted lines). This attests to the central role of evaporation in creating robust ΔT.

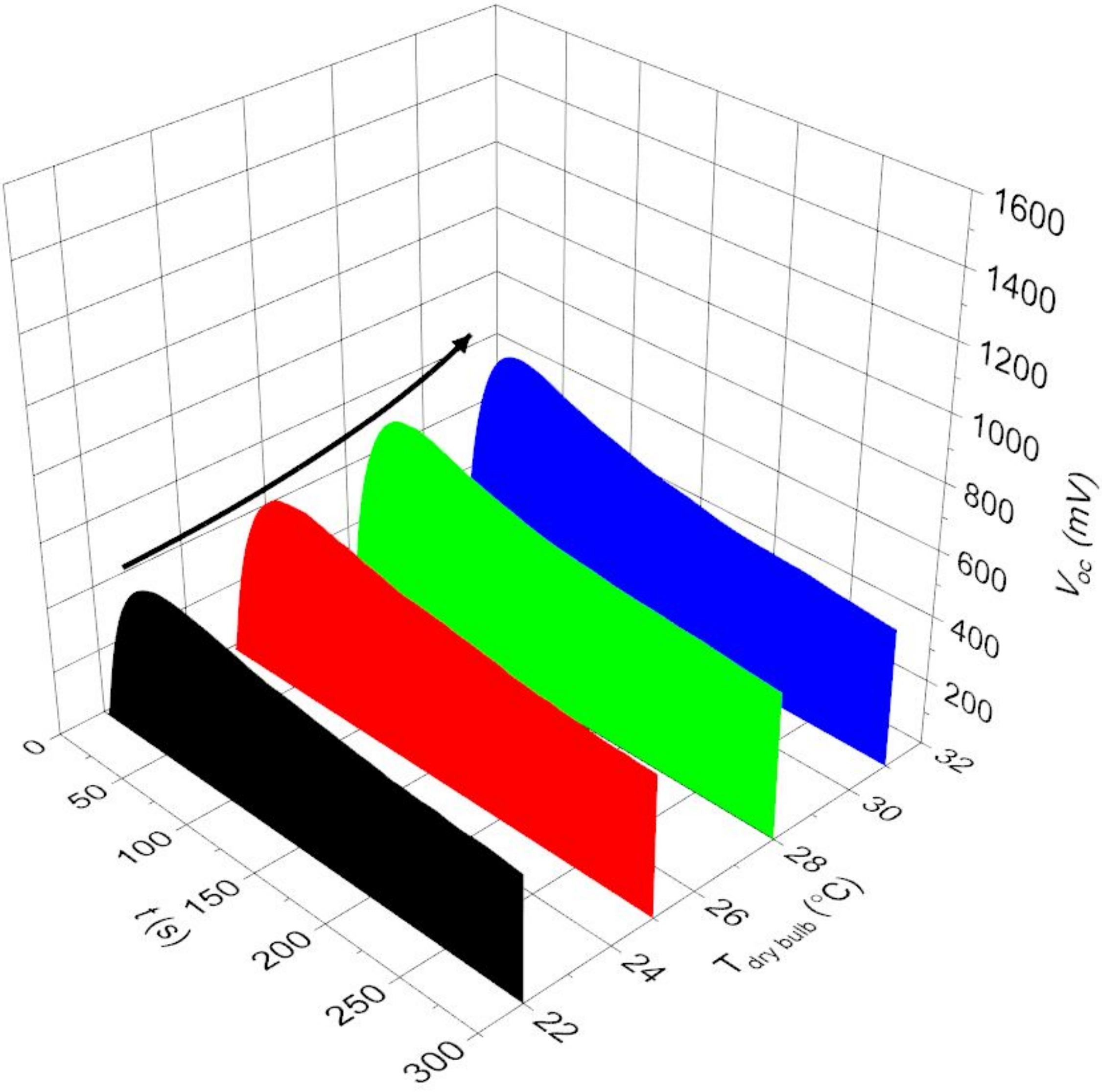

Fig. S11. Open-circuit voltage (V_{OC}) at 80% relative humidity (RH) as a function of time and $T_{dry\text{-}bulb}$.

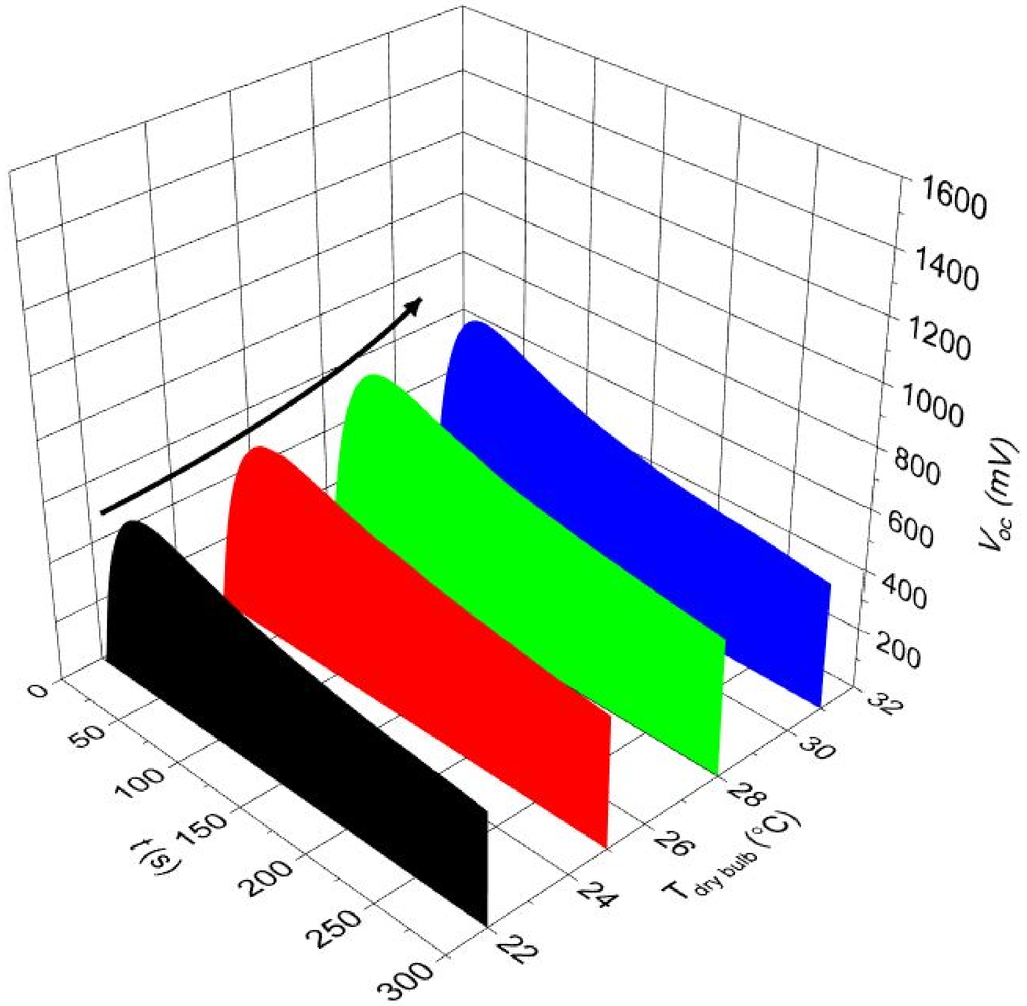

Fig. S12. Open-circuit voltage (V_{OC}) at 75% relative humidity (RH) as a function of time and $T_{dry\text{-}bulb}$.

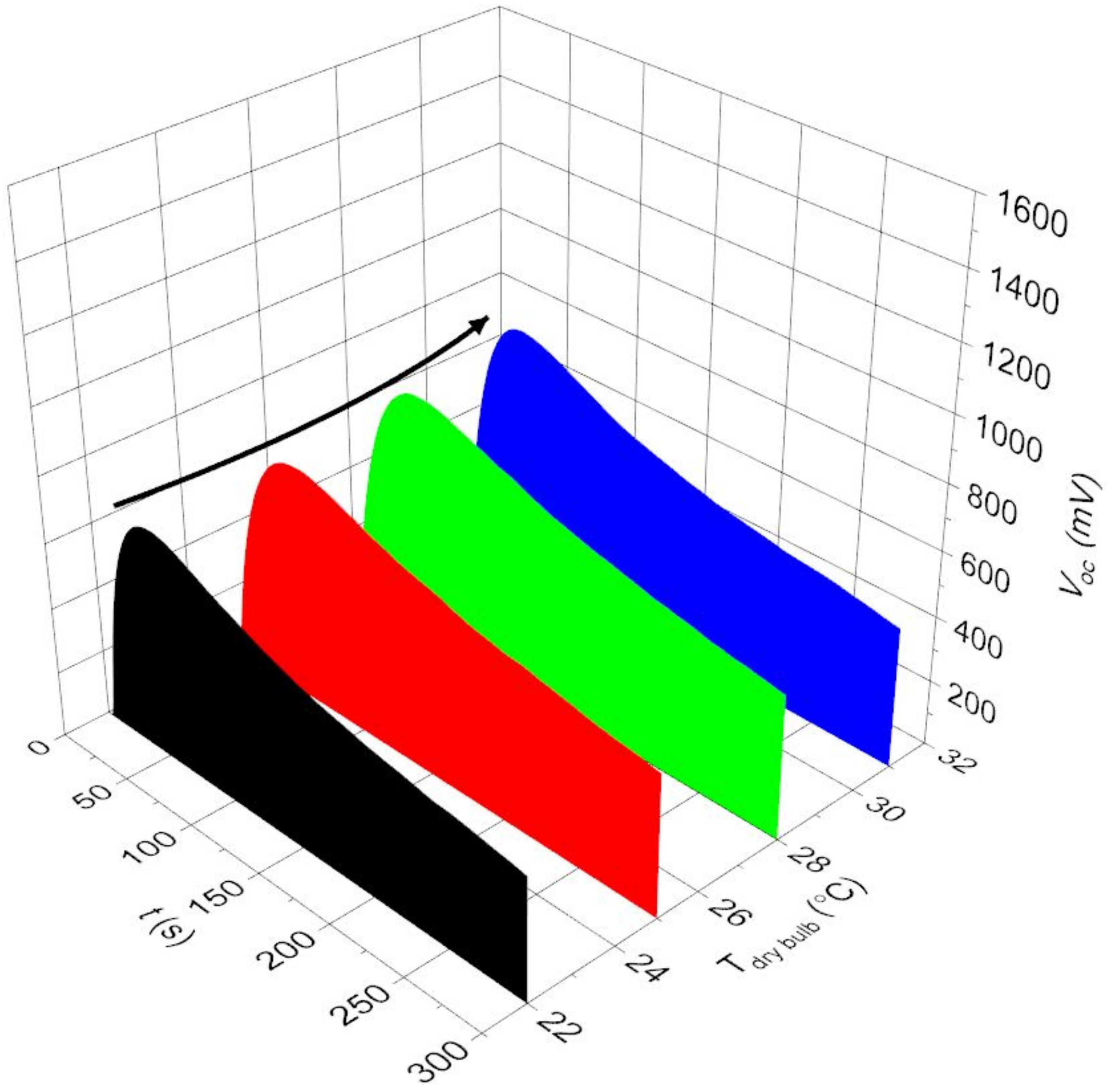

Fig. S13. Open-circuit voltage (V_{OC}) at 70% relative humidity (RH) as a function of time and $T_{dry\text{-}bulb}$.

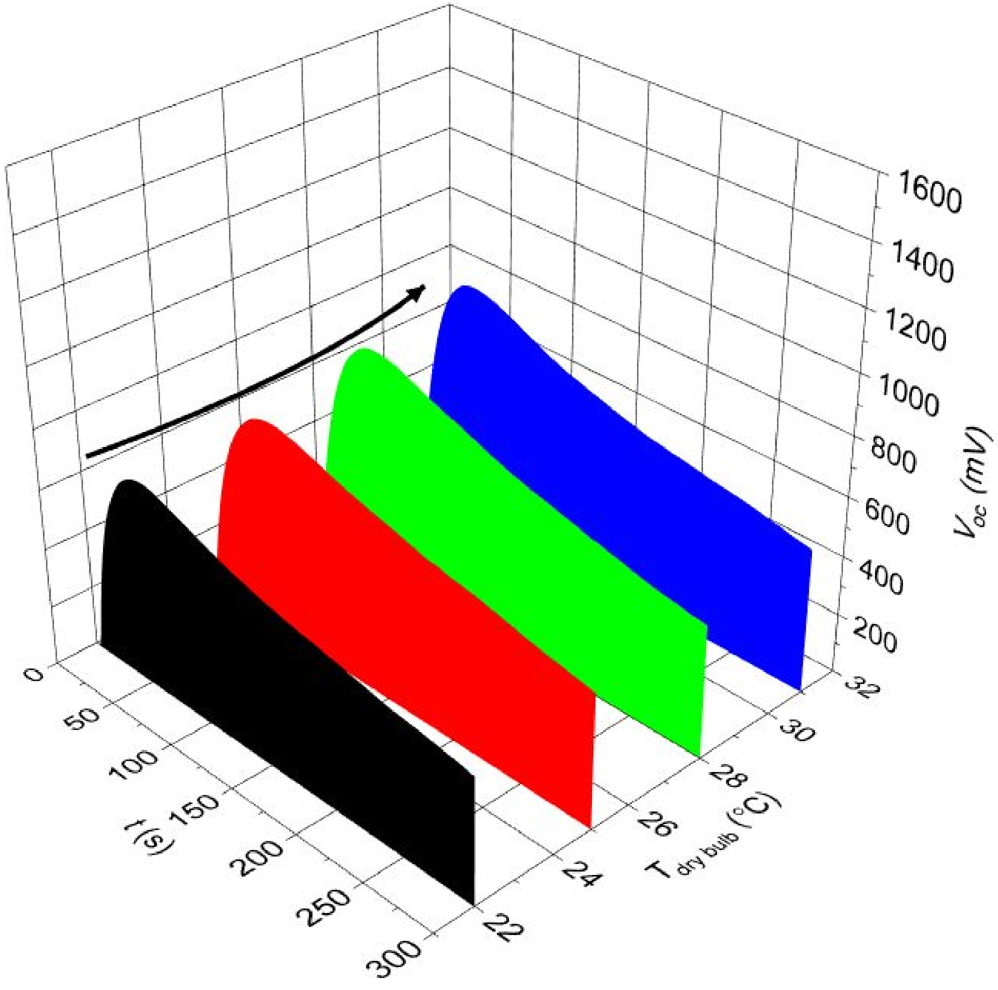

Fig. S14. Open-circuit voltage (V_{OC}) at 65% relative humidity (RH) as a function of time and $T_{dry\text{-}bulb}$.

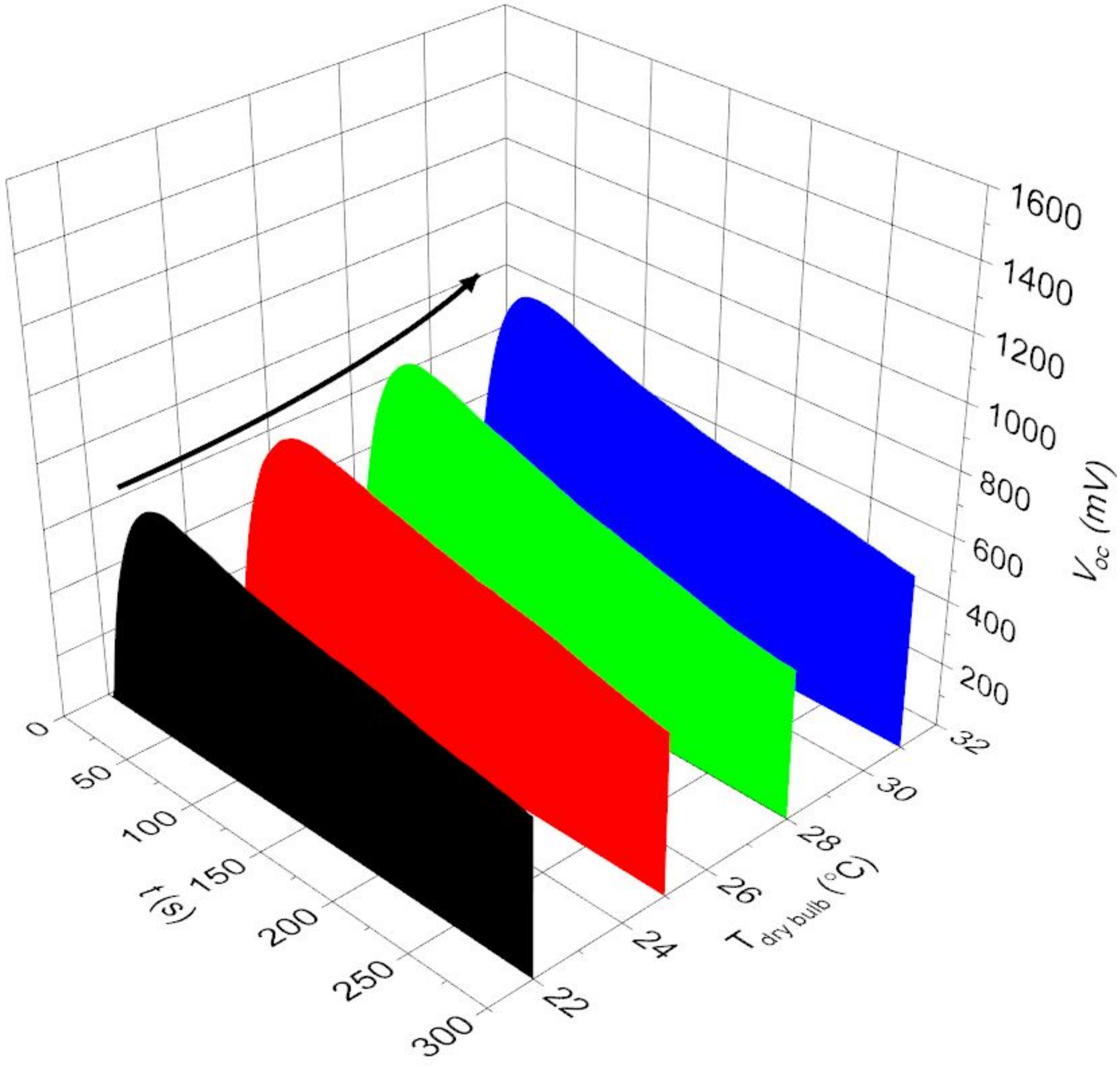

Fig. S15. Open-circuit voltage (V_{OC}) at 60% relative humidity (RH) as a function of time and $T_{dry\text{-}bulb}$.

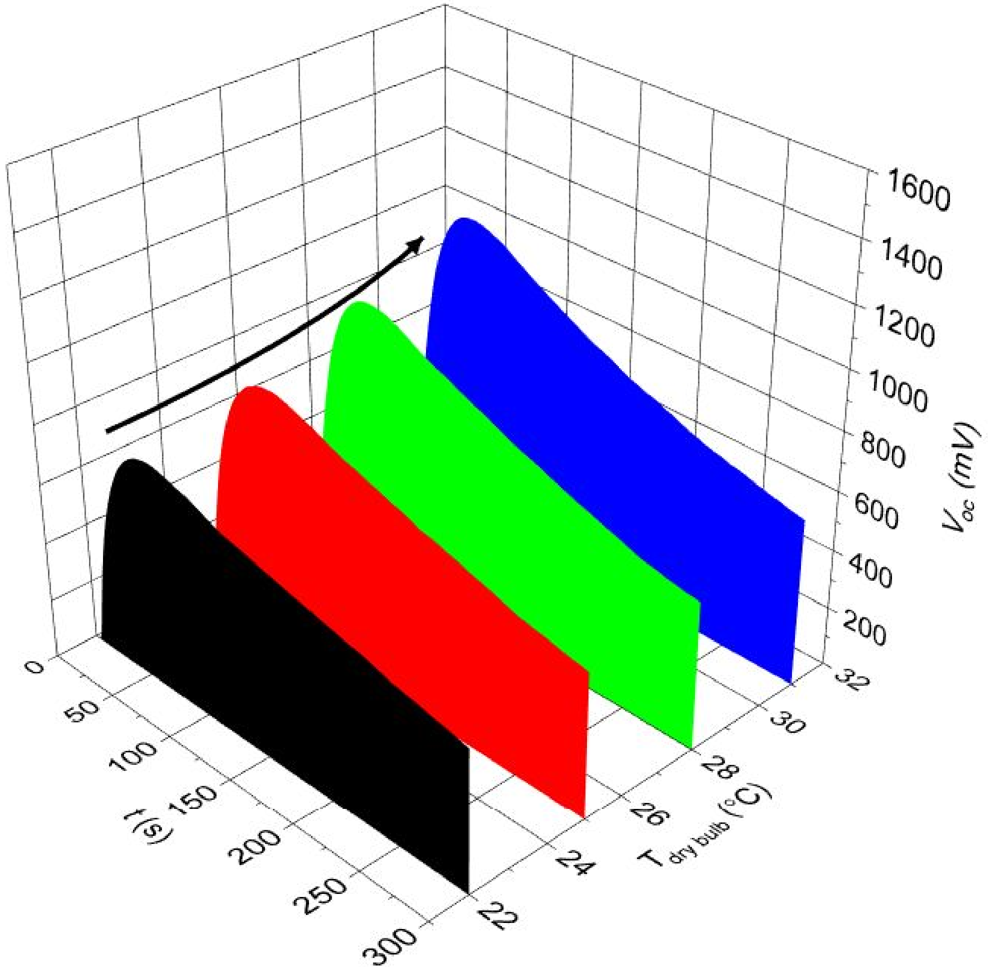

Fig. S16. Open-circuit voltage (V_{OC}) at 55% relative humidity (RH) as a function of time and $T_{dry\text{-}bulb}$.

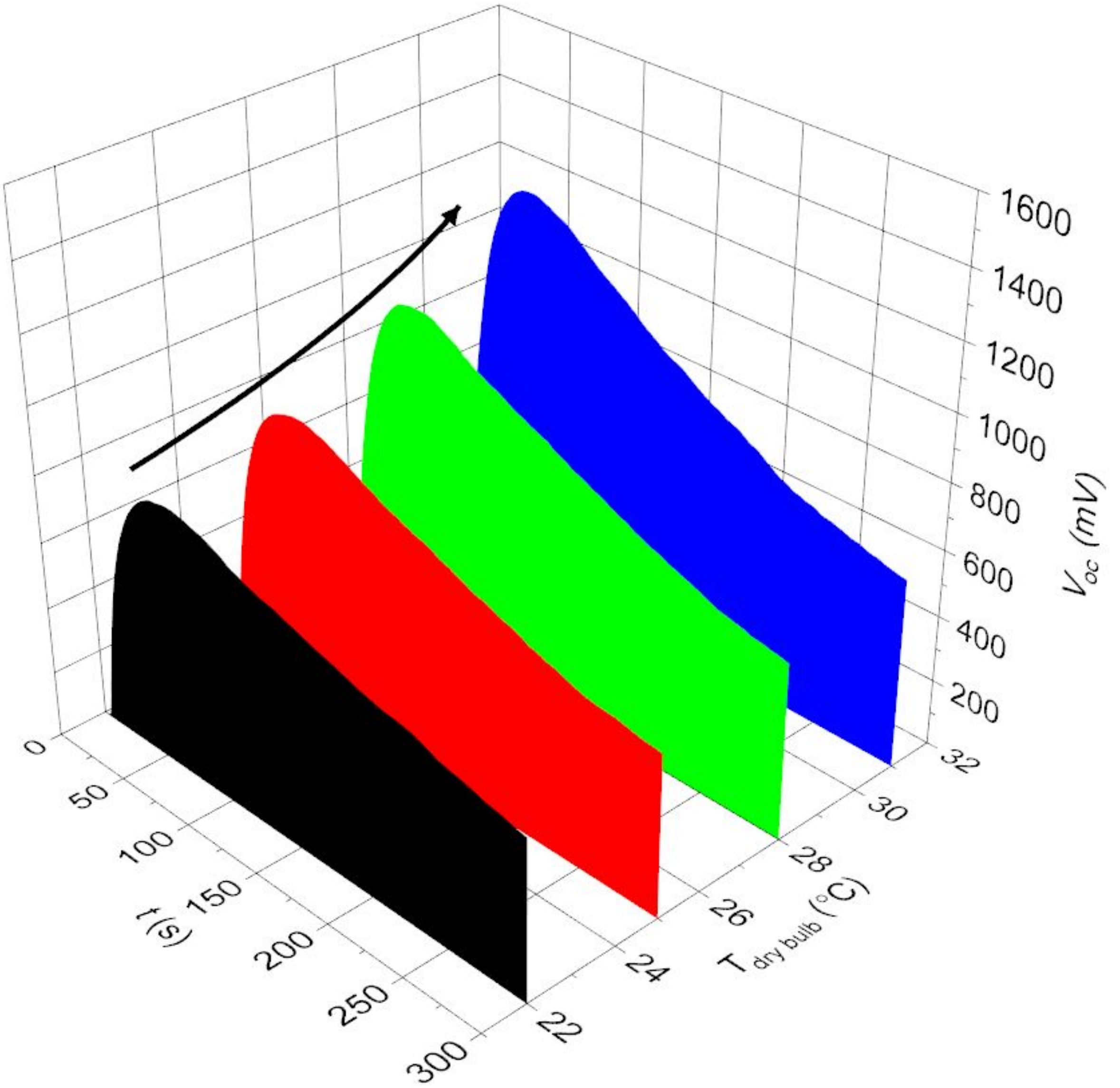

Fig. S17. Open-circuit voltage (V_{OC}) at 50% relative humidity (RH) as a function of time and $T_{dry\text{-}bulb}$.

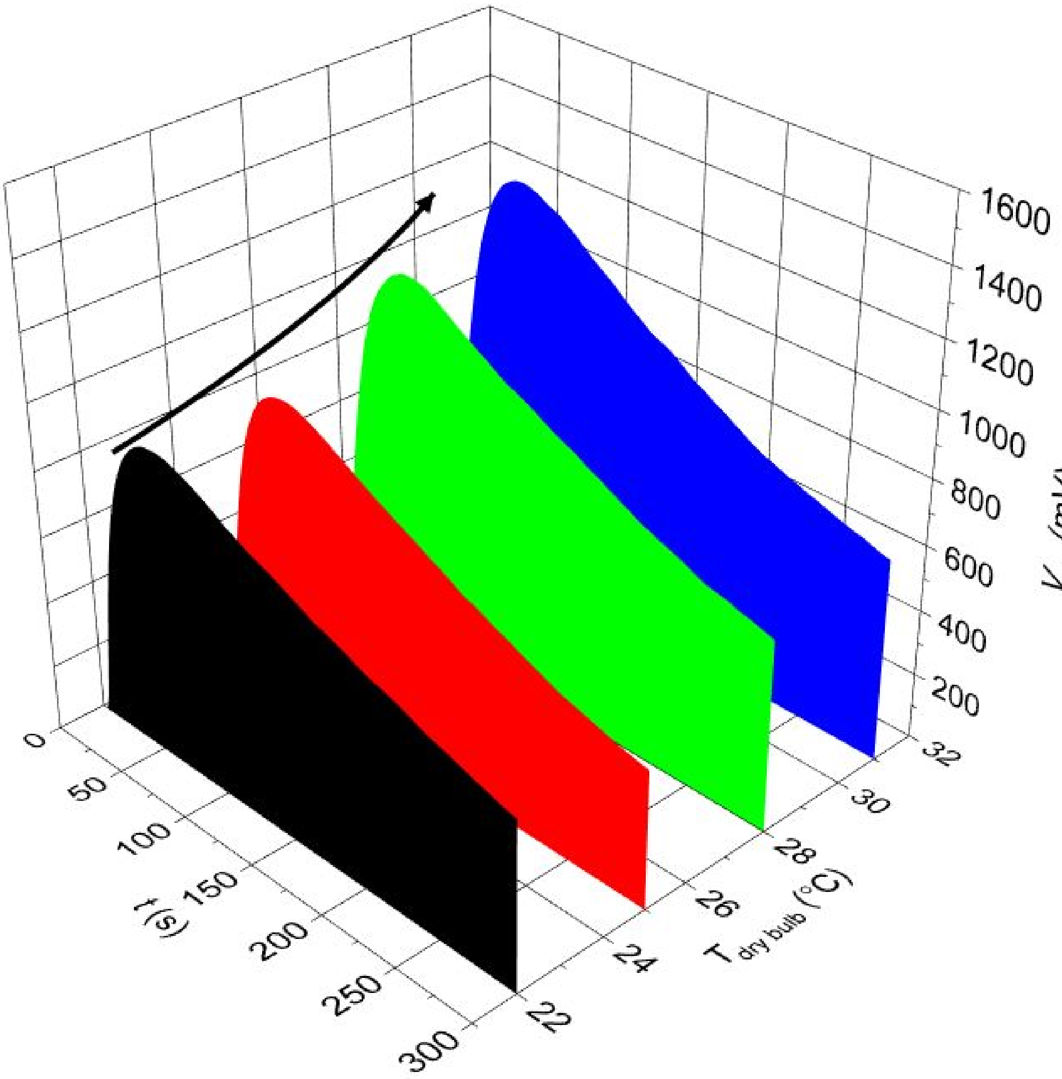

Fig. S18. Open-circuit voltage (V_{OC}) at 45% relative humidity (RH) as a function of time and $T_{dry-bulb}$.

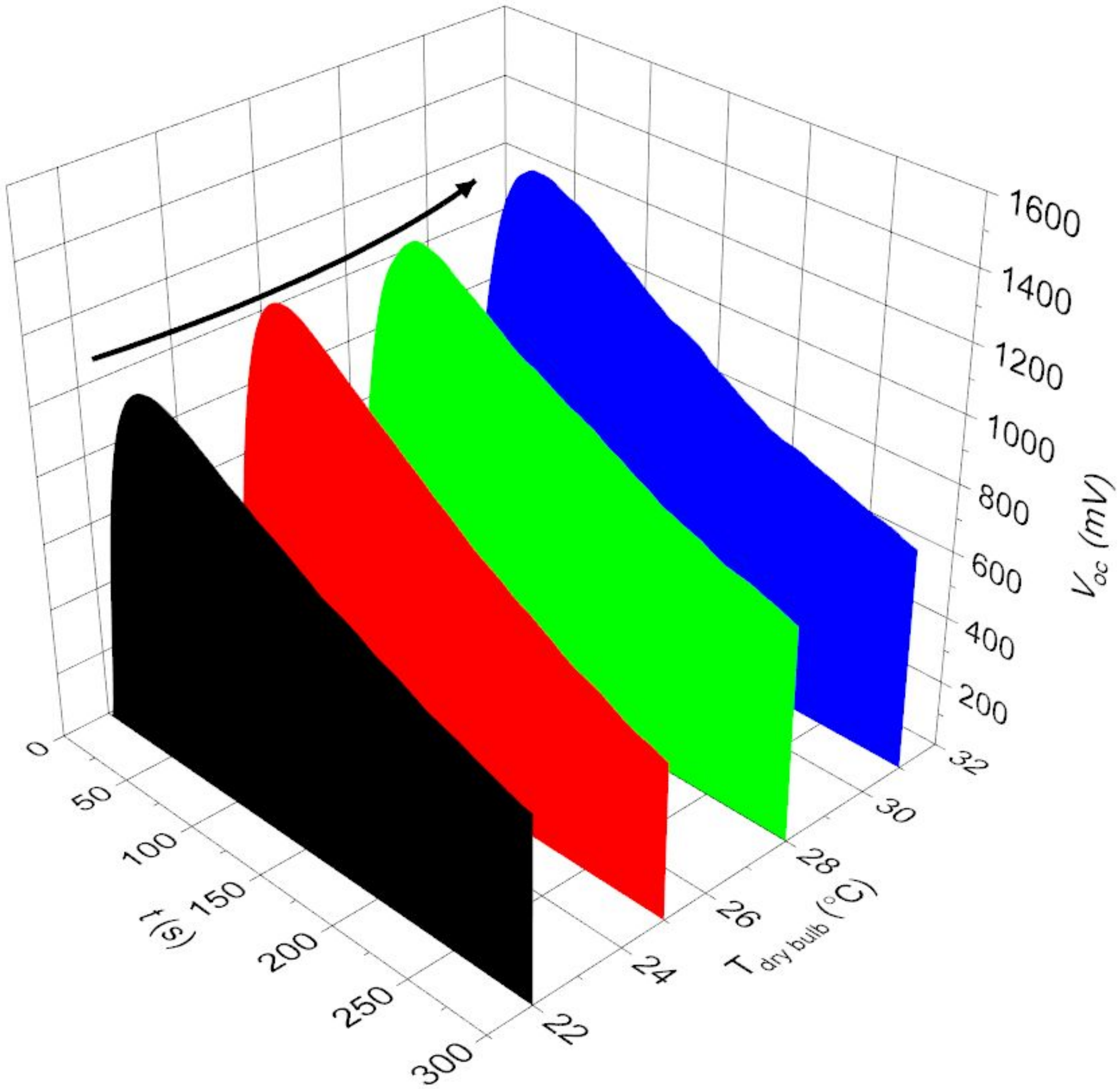

Fig. S19. Open-circuit voltage (V_{OC}) at 40% relative humidity (RH) as a function of time and $T_{dry\text{-}bulb}$.

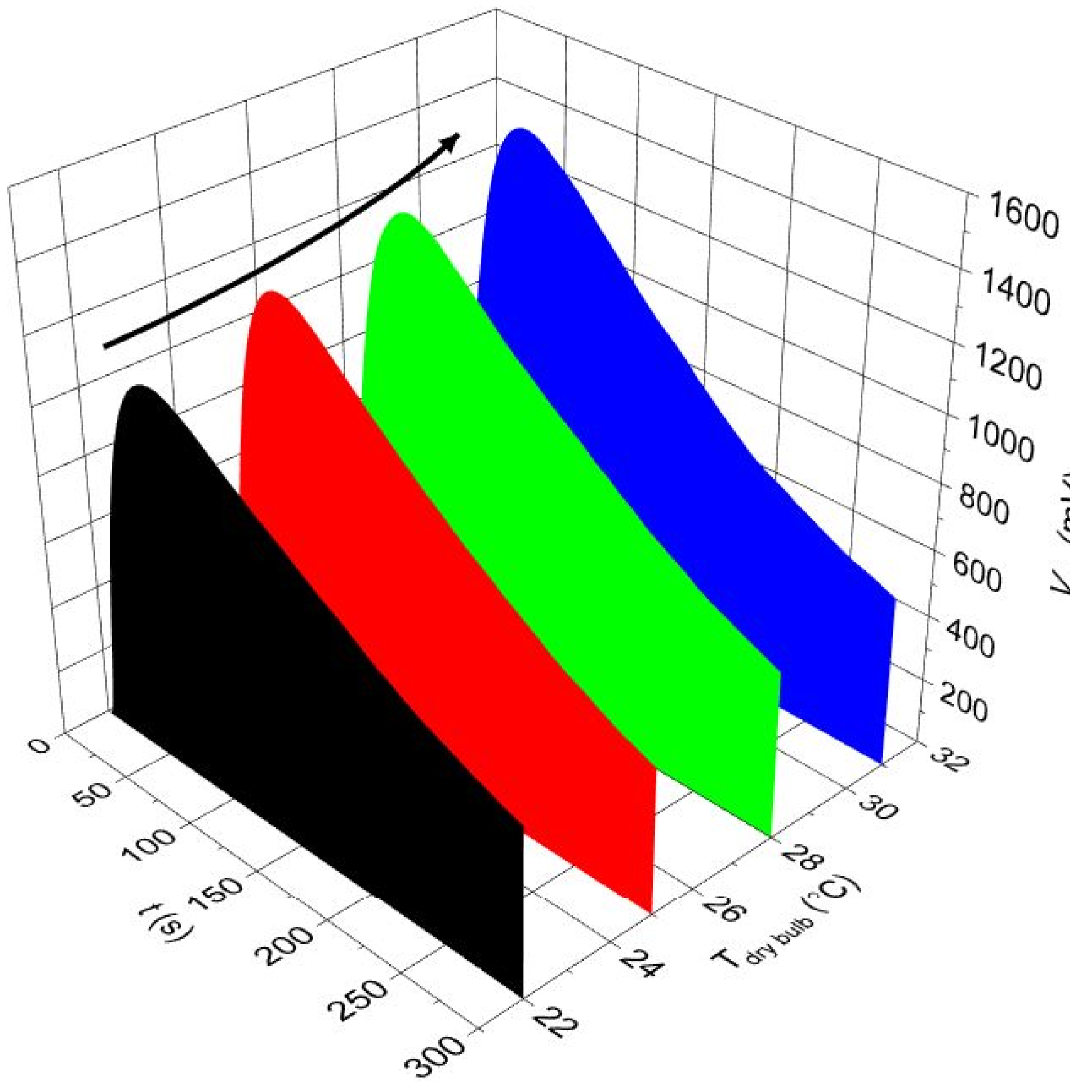

Fig. S20. Open-circuit voltage (V_{OC}) at 35% relative humidity (RH) as a function of time and $T_{dry\text{-}bulb.}$

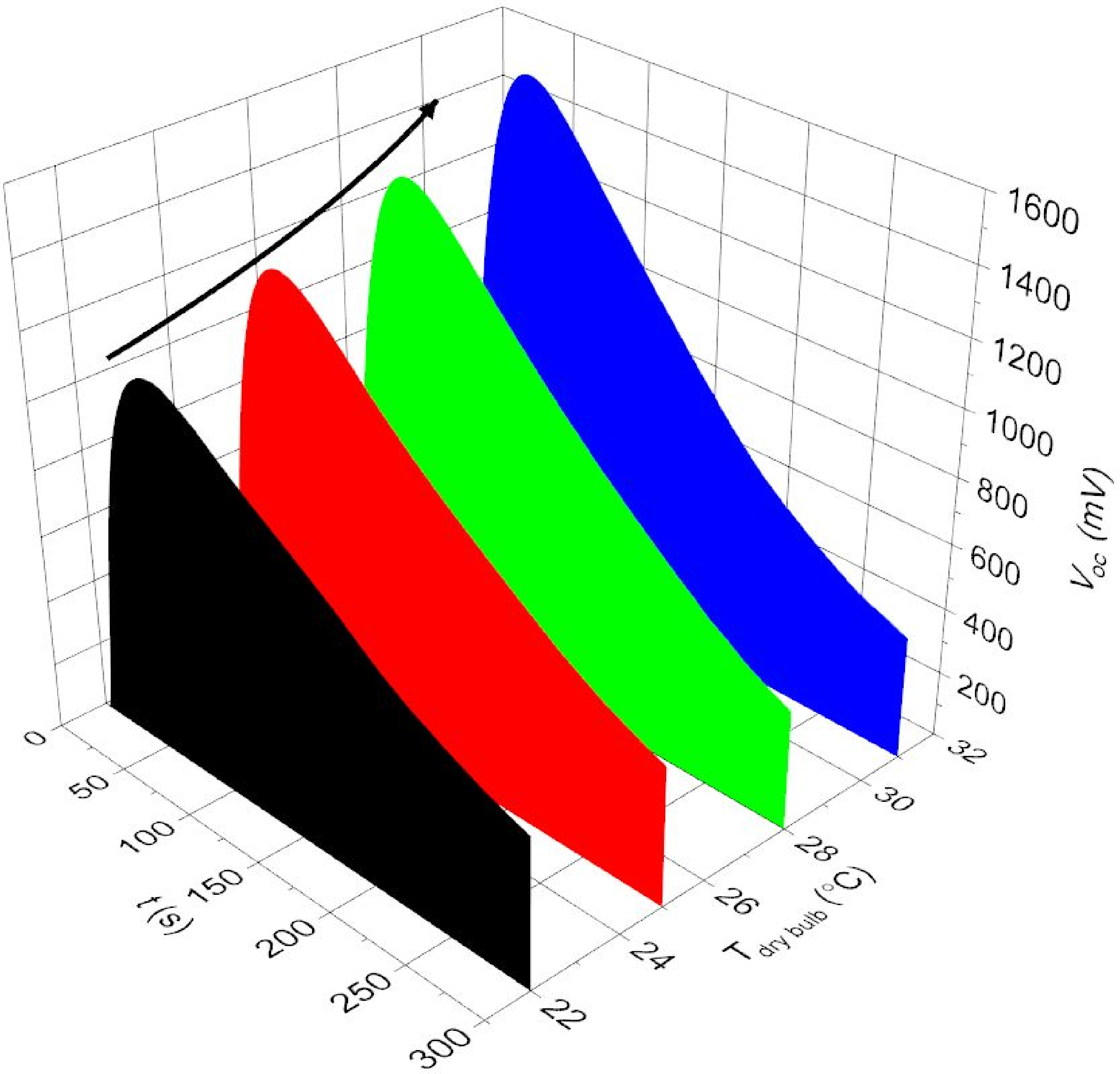

Fig. S21. Open-circuit voltage (V_{OC}) at 30% relative humidity (RH) as a function of time and $T_{dry\text{-}bulb}$.

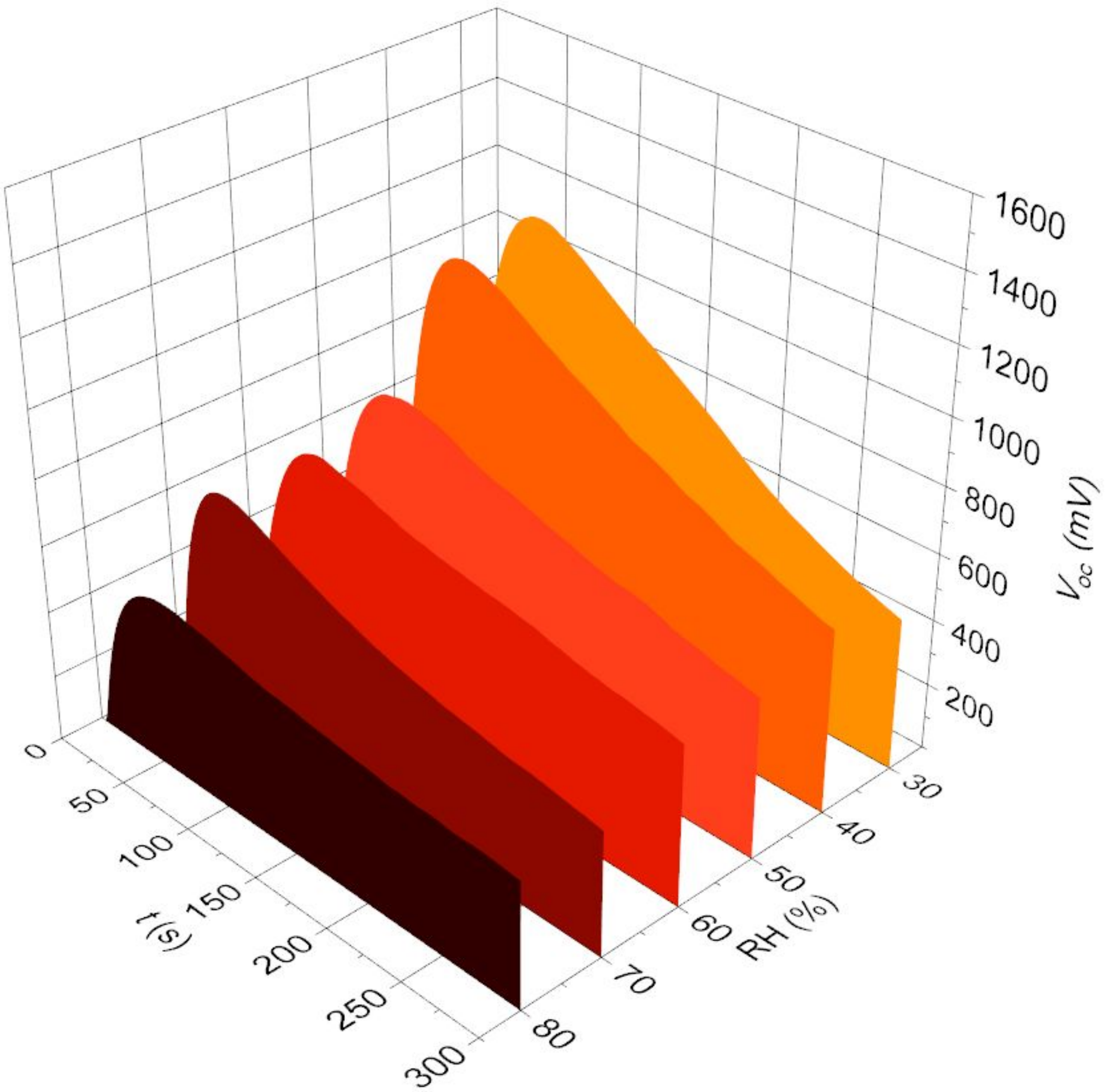

Fig. S22. Open-circuit voltage (V_{OC}) at $T_{dry\text{-}bulb}$ of 22 ℃ as a function of time and relative humidity (RH).

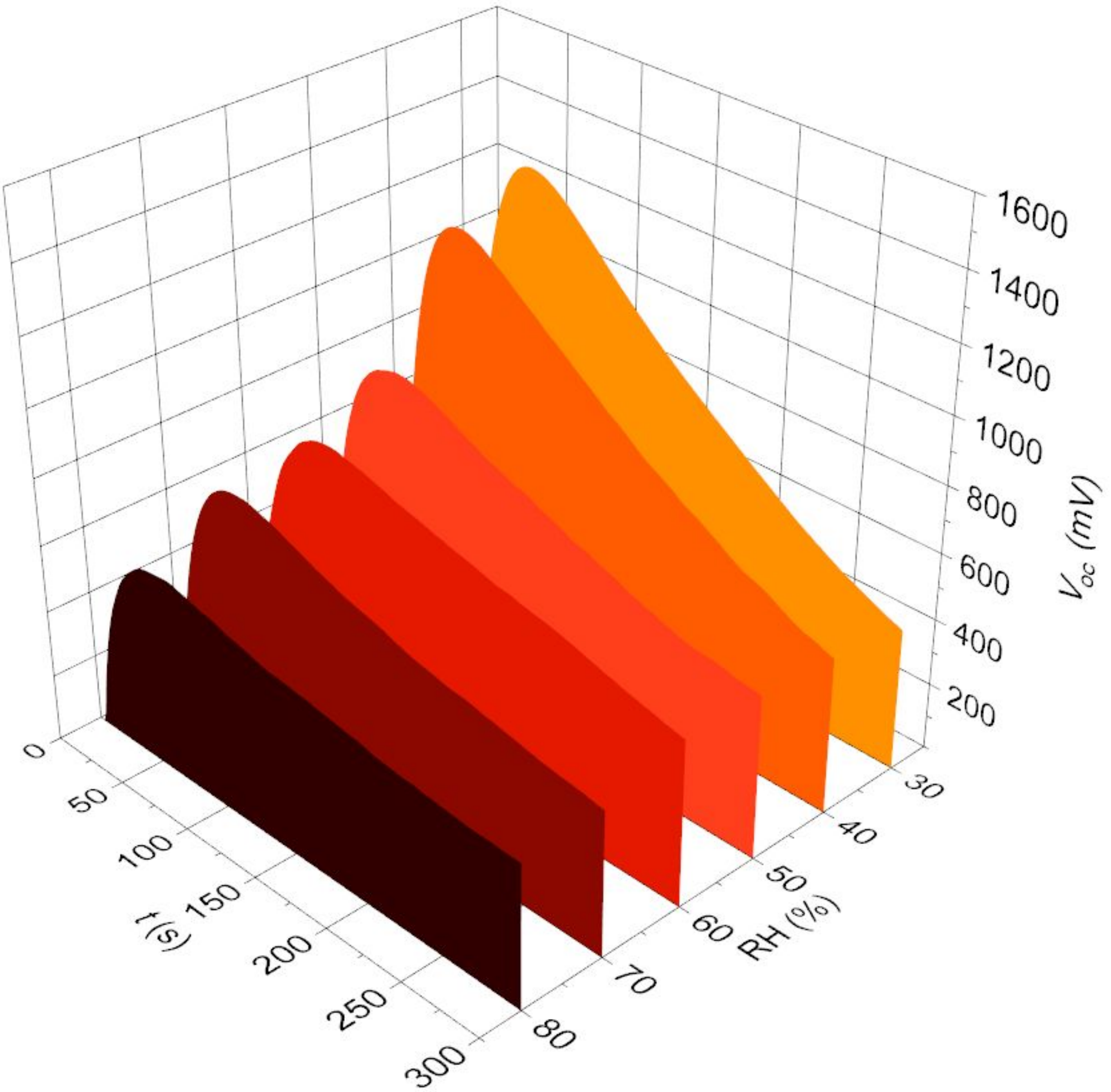

Fig. S23. Open-circuit voltage (V_{OC}) at $T_{dry\text{-}bulb}$ of 25 ℃ as a function of time and relative humidity (RH).

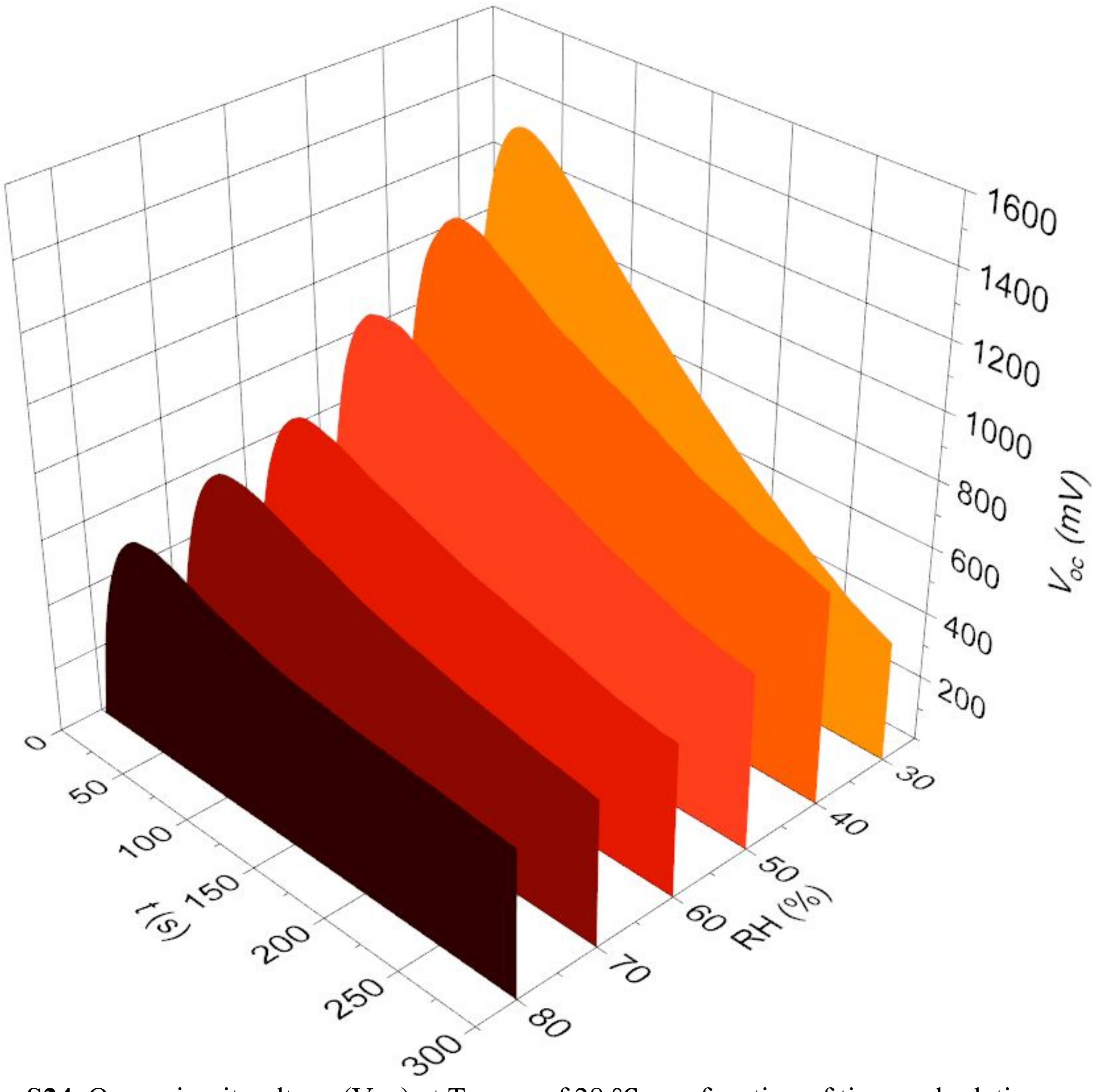

Fig. S24. Open-circuit voltage (V_{OC}) at $T_{dry\text{-}bulb}$ of 28 ℃ as a function of time and relative humidity (RH).

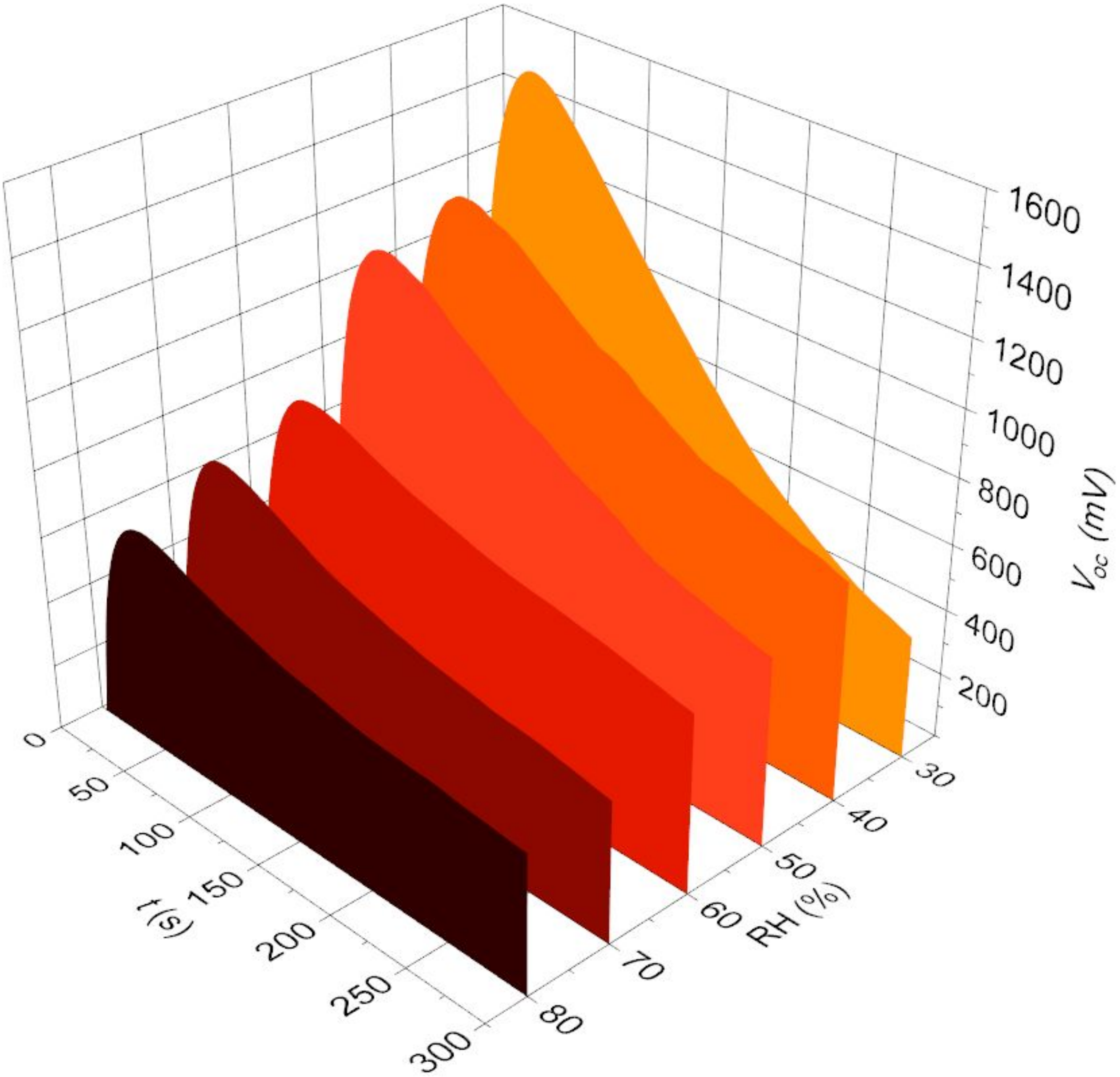

Fig. S25. Open-circuit voltage (V_{OC}) at $T_{dry\text{-}bulb}$ of 28 °C as a function of time and relative humidity (RH).

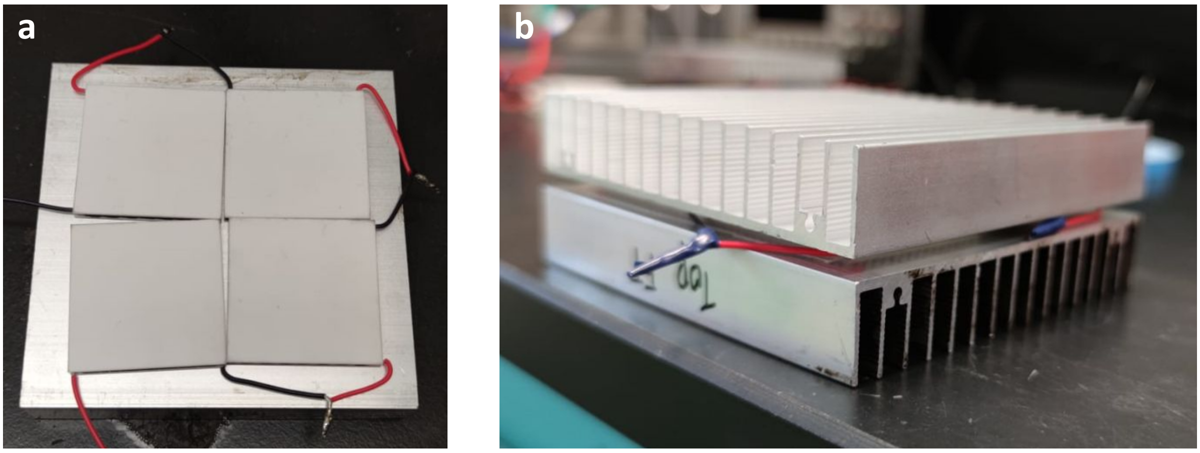

Fig. S26. Photo of evapolectric device. (a) 4 TEG modules were used in an evapolectric device. **(b)** TEG modules sandwiched between two perpendicularly aligned aluminum heat sinks.

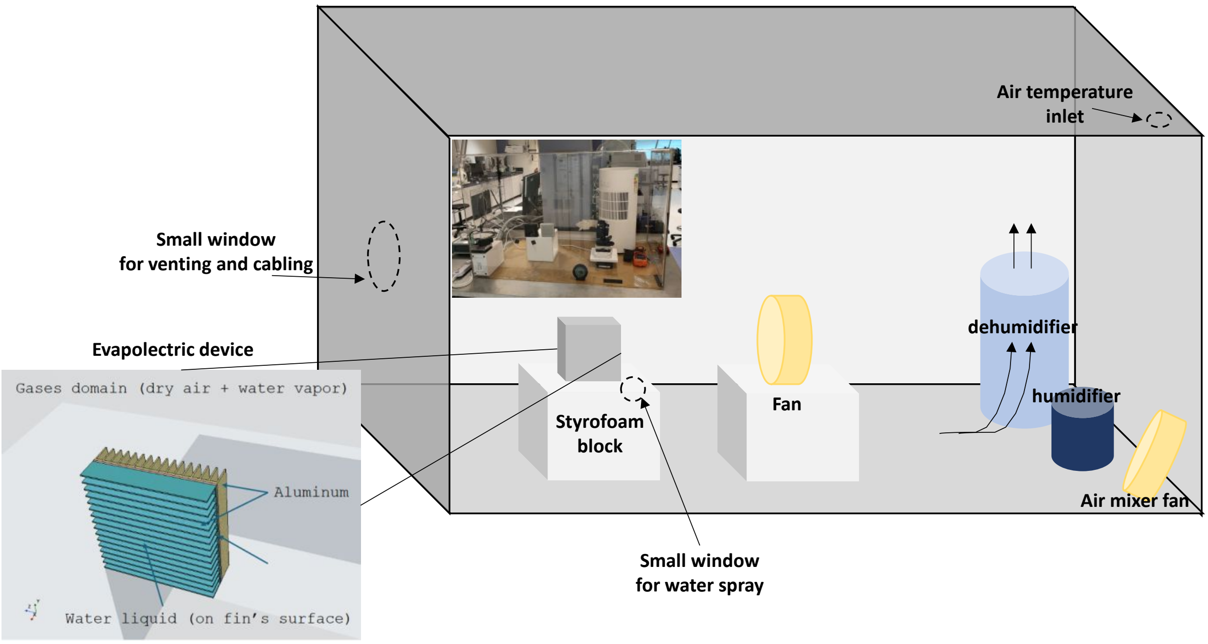

Fig. S27. Schematic of controlled environment setup for evapolectrics testing under different relative humidity and temperature.

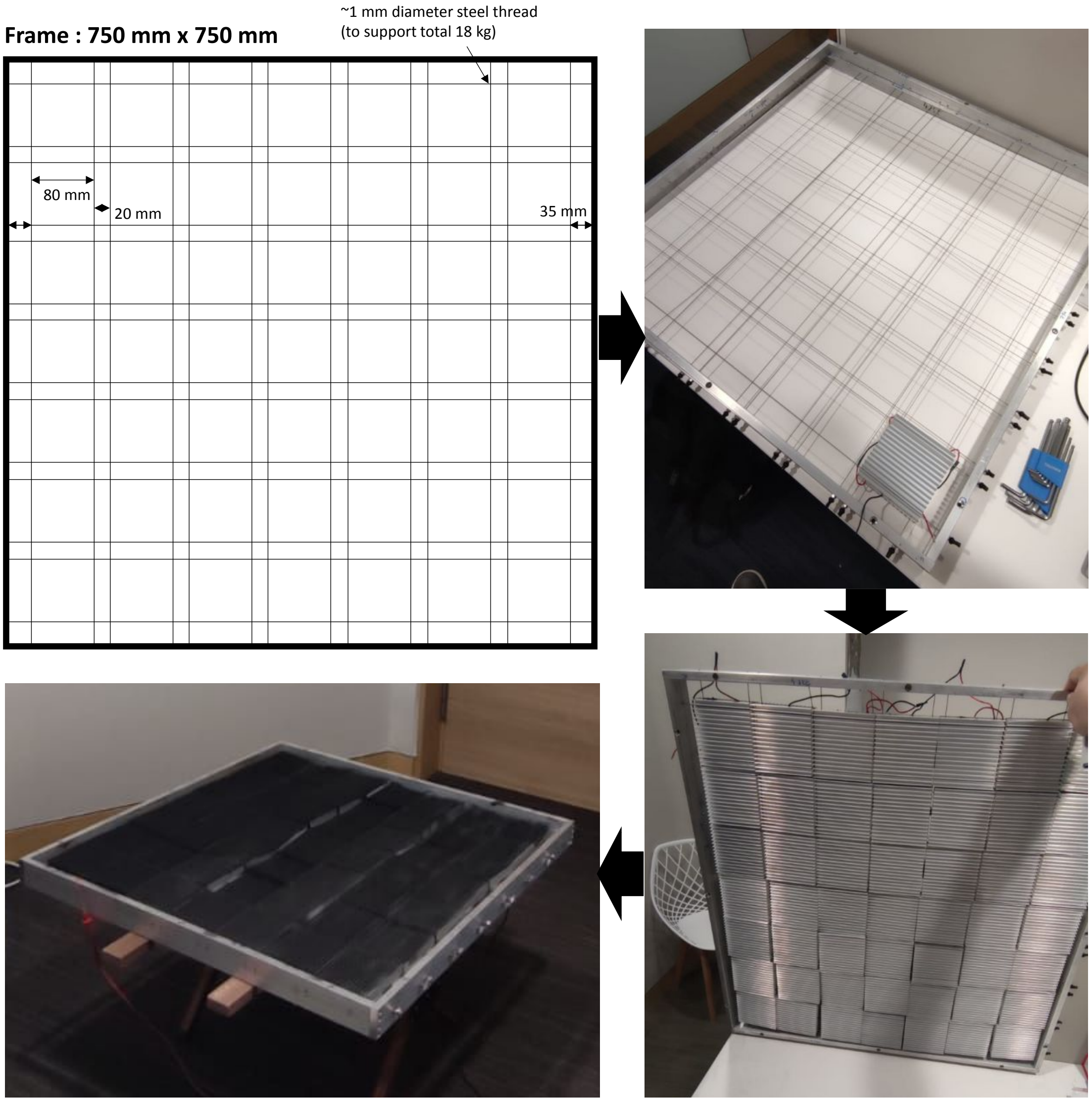

Fig. S28. 7 x 7 array of evapolectrics devices, and the holder design process.

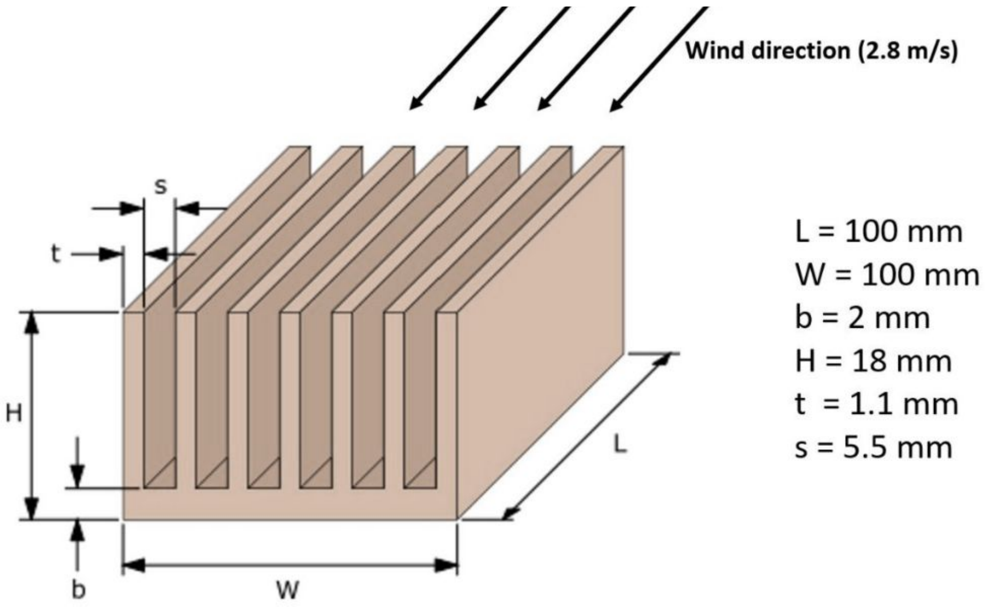

Fig. S29. Physical dimension of the heat sink used throughout the evapolectrics experiments.

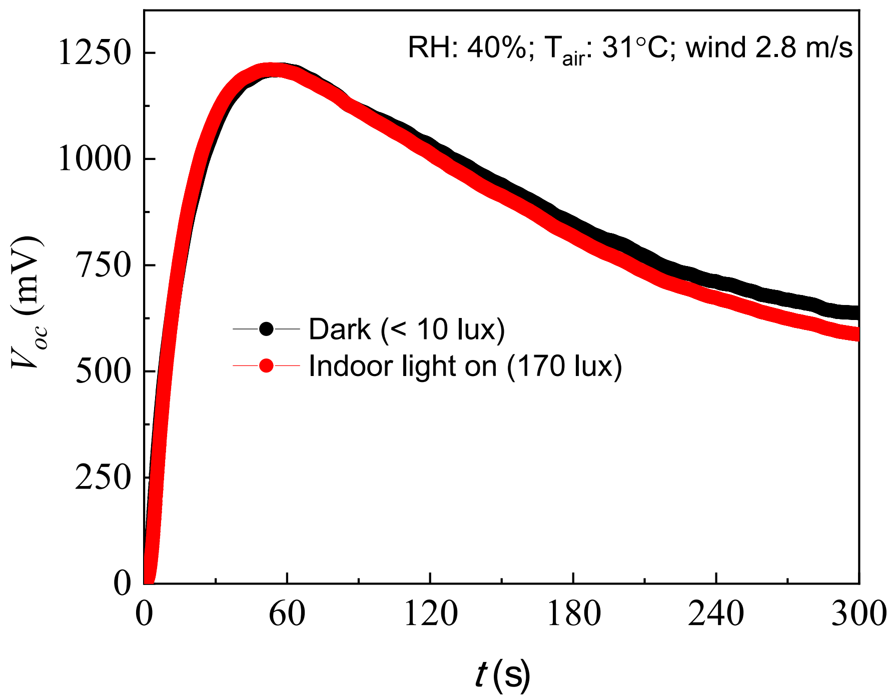

Fig. S30. Open circuit voltage profile of evapolectrics under dark condition vs indoor ambient lighting.

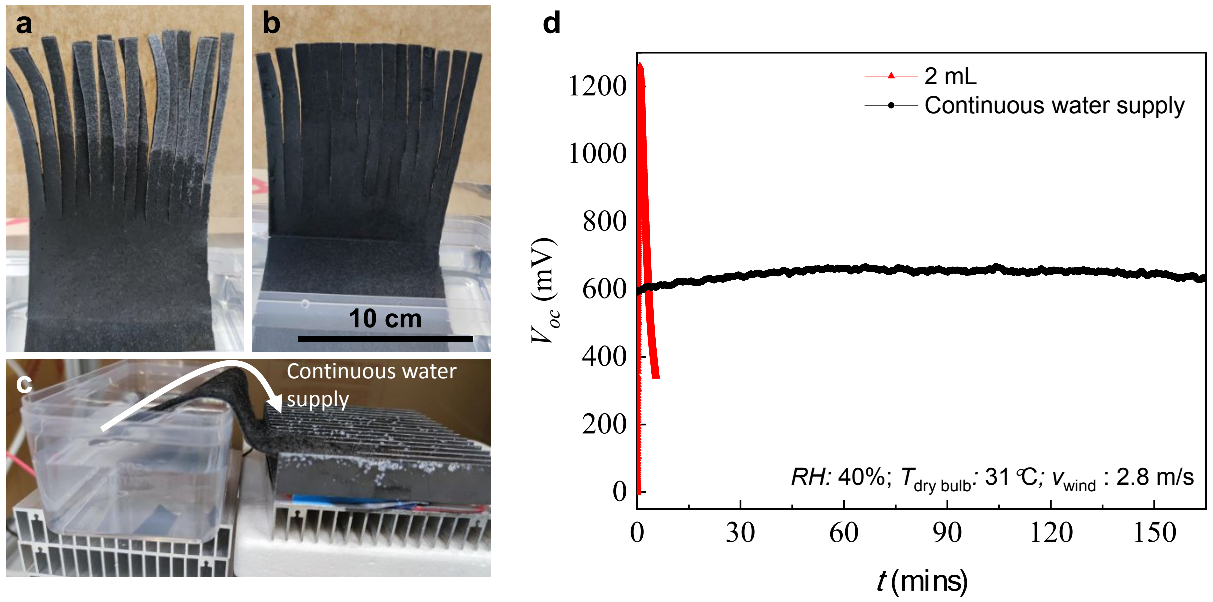

Fig. S31.

Setup for continuous water supply and evapolectrics performance showing (a) capillary in action as water is absorbed from reservoir by the capillary mat. (b) good water absorption (>10 cm) by the capillary mat. (c) experimental setup with continuous water supply from reservoir. (d) comparison of open circuit voltage between periodic spray (red) and continuous water supply (black).

Table S1. Experimental parameters and boundary conditions used in the CFD simulation.

**Parameter**	**Value**
Dynamic viscosity (air)	1.85508 x 10^{-5} Pa.s
Molecular weight (air)	28.9664 Kg/KMol
Specific heat (air)	1003.62 J/KgK
Dynamic viscosity (water vapor)	1.26765 x 10^{-5} Pa.s
Molecular weight (water vapor)	18.0153 Kg/Kmol
Heat of formation (water vapor)	-1.34234 x 10^{7} J/kg
Specific heat (water vapor)	1938.19 J/KgK
Density (water liquid)	997.561 Kg/m^3
Dynamic Viscosity (water liquid)	8.8871x10^{-4} Pa.s
Heat of formation (water liquid)	-1.5866 x 10^{7} J/kg
Latent heat of vaporization (water liquid)	2.4426 x 10^{6} KJ/Kg
Molecular weight (water liquid)	18.0153 Kg/Kmol
Specific heat (water liquid)	4181.72 J/KgK
Thermal conductivity (water liquid)	0.620271 W/(m.K)
Density (Aluminum)	2700 Kg/m^3
Specific heat (Aluminum)	900 J/KgK
Thermal conductivity (Aluminum)	205 W/(m.K)
Thermal conductivity (TEG)	1.5 W/(m.K)
Ambient temperature	30 ℃
Initial and ambient humidity	50 %
Initial heat sink fins and water temperature	30 ℃
Initial water liquid film thicknesses	41.67 µm (based on 2 ml uniform spreading of water). Also consistent with actual graphite thickness of 40 – 50 µm.

Table S2. Comparison in terms of energy input between evapolectrics and other evaporation-based energy harvesting technologies.

Reference	light source	Heat source	Electricity input	Application	Remarks
This work	○	○	○	Evapolectrics	RH has significant impact on output power
8-13	○	✓	○	Evaporation driven Thermoelectric waste heat recovery	RH was found to have *small impact* on output power
14-17	○	○	✓	Cooling device	Thermoelectric assisted evaporative cooling
18-35	✓	○	○	Solar-driven evaporation	Simultaneous evaporation and energy harvesting
36	✓	○	○	Solar-driven steam energy harvesting	Novel utilization of steam enthalpy during condensation (opposite of evapolectrics)
37-42	✓	○	○	Hydrovoltaics	Solar assisted hydrovoltaics
3, 4, 43-65	○	○	○	Hydrovoltaics	Electricity generation through streaming potential of capillary water movement, ion movement, or mechanical energy.

○ Not required

✓ Required

Table S3. Comparison among evaporation ambient energy harvesters including hydrovoltaics and photothermal.

Reference	Power density (mW/m^2)
This work	4200
3	6.75
4	0.93
43	263
44	0.35
66	165
67	0.14
56	0.53
68	98.1
69	24.5
70	2.5
47	13.5
48	101
51	640*
4	250
54	389
71	10
61	50
72	120
73	460
74	400
75	187.4
76	15.77
77	250
78	0.196
79	6850
19	5550

*Assuming fill factor of 0.25 for commercial TEG (thermoelectric generator)

Supplementary Video S1.

Computational fluid dynamics (CFD) simulation showing the time evolution of surrounding air temperature and front face heat sink temperature as the water evaporates. Wind direction and gravity orientation are indicated by respective arrows.

Supplementary Video S2.

Computational fluid dynamics (CFD) simulation showing the time evolution of mass-fraction of water in the surrounding air and at the front face heat sink as the water evaporates. Wind direction and gravity orientation are indicated by respective arrows.

Supplementary Video S3.

7 x 7 array of evapolectrics sprinkled with water showing enough power generated from evaporation to charge smartphone.

Supplementary Video S4.

Thermal imaging video showing the side by side comparison of time evolution of temperature between non-coated and graphite-coated heat sinks.

Supplementary Video S5.

Real-time LabVIEW video showing the data acquisition of V_{OC} (open-circuit voltage) after water is introduced onto heat sink surface. Video is fast forwarded after 40s.

Supplementary Video S6.

Real-time LabVIEW video showing the output power vs load resistance after water is introduced onto heat sink surface.

References

1. Zhang, Y., Thermophysical interpretation of the latent heat of vaporization of water. *International journal of thermophysics* **1989,** *10*, 911-915.
2. Chen, X.; Mahadevan, L.; Driks, A.; Sahin, O., Bacillus spores as building blocks for stimuli-responsive materials and nanogenerators. *Nature nanotechnology* **2014,** *9* (2), 137-141.
3. He, N.; Wang, H.; Li, F.; Jiang, B.; Tang, D.; Li, L., Ion engines in hydrogels boosting hydrovoltaic electricity generation. *Energy & Environmental Science* **2023,** *16* (6), 2494-2504.
4. Yu, F.; Li, J.; Jiang, Y.; Wang, L.; Yang, X.; Yang, Y.; Li, X.; Jiang, K.; Lü, W.; Sun, X., High Hydrovoltaic Power Density Achieved by Universal Evaporating Potential Devices. *Advanced Science* **2023,** *10* (30), 2302941.
5. Meng, S.; Tang, C. Y.; Yang, J.; Yang, M. B.; Yang, W., A Wave-Driven Piezoelectrical Film for Interfacial Steam Generation: Beyond the Limitation of Hydrogel. *Advanced Science* **2022,** *9* (33), 2204187.
6. Liu, H.; Luo, H.; Huang, J.; Chen, Z.; Yu, Z.; Lai, Y., Programmable Water/Light Dual-Responsive Hollow Hydrogel Fiber Actuator for Efficient Desalination with Anti-Salt Accumulation. *Advanced Functional Materials* **2023,** *33* (33), 2302038.
7. Chaule, S.; Kang, J.; Jang, J.-H., Optimizing solar evaporation efficiency: integrating controllable water supply and efficient salt collection methods. *Materials Today Energy* **2024,** *43*, 101588.
8. Zheng, L. J.; Kang, H. W., A passive evaporative cooling heat sink method for enhancing low-grade waste heat recovery capacity of thermoelectric generators. *Energy Conversion and Management* **2022,** *251*, 114931.
9. Zheng, L. J.; Lim, S.; Kim, N. K.; Kang, D. H.; Youn, Y. J.; Lee, W.; Kang, H. W., Experimental study of a thin water-film evaporative cooling system to enhance the energy conversion efficiency of a thermoelectric device. *Energy* **2020,** *211*, 119040.
10. Zheng, L. J.; Kang, D. H.; Kim, N. K.; Youn, Y. J.; Kang, H. W., Theoretical analysis of natural evaporative cooling to enhance the efficiency of thermoelectric devices. *International Journal of Heat and Mass Transfer* **2019,** *143*, 118512.
11. Gao, Y.; Wu, D.; Dai, Z.; Wang, C.; Zhu, L.; Zhang, J.; Xu, G.; Zhang, X., A passive evaporative cooling strategy to enhance the electricity production of hybrid PV-STEG system. *Applied Energy* **2023,** *349*, 121689.
12. Poddar, V. S.; Ranawade, V. A.; Dhokey, N. B., Study of synergy between photovoltaic, thermoelectric and direct evaporative cooling system for improved performance. *Renewable Energy* **2022,** *182*, 817-826.
13. Boonyasri, M.; Jamradloedluk, J.; Lertsatitthanakorn, C.; Therdyothin, A.; Soponronnarit, S., Increasing the Efficiency of a Thermoelectric Generator Using an Evaporative Cooling System. *Journal of Electronic Materials* **2017,** *46* (5), 3043-3048.
14. Zhou, Y.; Zhang, T.; Wang, F.; Yu, Y., Performance analysis of a novel thermoelectric assisted indirect evaporative cooling system. *Energy* **2018,** *162*, 299-308.
15. Zhou, Y.; Yan, Z.; Dai, Q.; Yu, Y., Experimental study on the performance of a novel hybrid indirect evaporative cooling/thermoelectric cooling system. *Building and Environment* **2022,** *207*, 108539.
16. Zhou, Y.; Yan, Z.; Dai, Q.; Yu, Y., Experimental and numerical evaluation of a two-stage indirect/thermoelectric assisted direct evaporative cooling system. *Energy Conversion and Management* **2021,** *248*, 114780.
17. Zhou, Y.; Zhang, T.; Wang, F.; Yu, Y., Numerical Study and Optimization of a Combined Thermoelectric Assisted Indirect Evaporative Cooling System. *Journal of Thermal Science* **2020,** *29* (5), 1345-1354.

18. Cui, Y.; Liu, J.; Li, Z.; Ji, M.; Zhao, M.; Shen, M.; Han, X.; Jia, T.; Li, C.; Wang, Y., Donor–acceptor-type organic-small-molecule-based solar-energy-absorbing material for highly efficient water evaporation and thermoelectric power generation. *Advanced Functional Materials* **2021,** *31* (49), 2106247.
19. Zeng, L.; Deng, D.; Zhu, L.; Zhang, Z.; Gu, X.; Wang, H.; Jiang, Y., Multi-scale CuS-rGO pyramidal photothermal structure for highly efficient solar-driven water evaporation and thermoelectric power generation. *Nano Energy* **2024,** *125*, 109531.
20. Zhu, L.; Ding, T.; Gao, M.; Peh, C. K. N.; Ho, G. W., Shape conformal and thermal insulative organic solar absorber sponge for photothermal water evaporation and thermoelectric power generation. *Advanced Energy Materials* **2019,** *9* (22), 1900250.
21. Bai, B.-L.; Du, S.; Li, M.-J., Solar-driven photovoltaic-steam-thermoelectric-steam cogeneration system by the interfacial cooling design. *Energy Conversion and Management* **2024,** *302*, 118147.
22. Wang, W.; Shi, Y.; Zhang, C.; Hong, S.; Shi, L.; Chang, J.; Li, R.; Jin, Y.; Ong, C.; Zhuo, S., Simultaneous production of fresh water and electricity via multistage solar photovoltaic membrane distillation. *Nature communications* **2019,** *10* (1), 3012.
23. Huang, L.; Jiang, H.; Wang, Y.; Ouyang, Z.; Wang, W.; Yang, B.; Liu, H.; Hu, X., Enhanced water yield of solar desalination by thermal concentrated multistage distiller. *Desalination* **2020,** *477*, 114260.
24. Yang, L.; Sun, T.; Tang, J.; Shao, Y.; Li, N.; Shen, A.; Chen, J.; Zhang, Y.; Liu, H.; Xue, G., Photovoltaic-multistage desalination of hypersaline waters for simultaneous electricity, water and salt harvesting via automatic rinsing. *Nano Energy* **2021,** *87*, 106163.
25. Zhu, L.; Gao, M.; Peh, C. K. N.; Wang, X.; Ho, G. W., Self-contained monolithic carbon sponges for solar-driven interfacial water evaporation distillation and electricity generation. *Advanced Energy Materials* **2018,** *8* (16), 1702149.
26. Cui, L.; Zhang, P.; Xiao, Y.; Liang, Y.; Liang, H.; Cheng, Z.; Qu, L., High rate production of clean water based on the combined photo-electro-thermal effect of graphene architecture. *Advanced materials* **2018,** *30* (22), 1706805.
27. Gao, M.; Peh, C. K.; Phan, H. T.; Zhu, L.; Ho, G. W., Solar absorber gel: localized macro-nano heat channeling for efficient plasmonic Au nanoflowers photothermic vaporization and triboelectric generation. *Advanced energy materials* **2018,** *8* (25), 1800711.
28. Xu, N.; Zhu, P.; Sheng, Y.; Zhou, L.; Li, X.; Tan, H.; Zhu, S.; Zhu, J., Synergistic tandem solar electricity-water generators. *Joule* **2020,** *4* (2), 347-358.
29. Yang, P.; Liu, K.; Chen, Q.; Li, J.; Duan, J.; Xue, G.; Xu, Z.; Xie, W.; Zhou, J., Solar-driven simultaneous steam production and electricity generation from salinity. *Energy & Environmental Science* **2017,** *10* (9), 1923-1927.
30. Zhang, Y.; Ravi, S. K.; Tan, S. C., Food-derived carbonaceous materials for solar desalination and thermo-electric power generation. *Nano Energy* **2019,** *65*, 104006.
31. Dao, V.-D.; Vu, N. H.; Choi, H.-S., All day Limnobium laevigatum inspired nanogenerator self-driven via water evaporation. *Journal of Power Sources* **2020,** *448*, 227388.
32. Hou, B.; Kong, D.; Qian, J.; Yu, Y.; Cui, Z.; Liu, X.; Wang, J.; Mei, T.; Li, J.; Wang, X., Flexible and portable graphene on carbon cloth as a power generator for electricity generation. *Carbon* **2018,** *140*, 488-493.
33. Dao, V.-D.; Vu, N. H.; Yun, S., Recent advances and challenges for solar-driven water evaporation system toward applications. *Nano Energy* **2020,** *68*, 104324.
34. Dao, V.-D.; Vu, N. H.; Dang, H.-L. T.; Yun, S., Recent advances and challenges for water evaporation-induced electricity toward applications. *Nano Energy* **2021,** *85*, 105979.
35. Dao, V.-D., An experimental exploration of generating electricity from nature-inspired hierarchical evaporator: The role of electrode materials. *Science of The Total Environment* **2021,** *759*, 143490.

36. Li, X.; Min, X.; Li, J.; Xu, N.; Zhu, P.; Zhu, B.; Zhu, S.; Zhu, J., Storage and recycling of interfacial solar steam enthalpy. *Joule* **2018,** *2* (11), 2477-2484.
37. Li, L.; Feng, S.; Bai, Y.; Yang, X.; Liu, M.; Hao, M.; Wang, S.; Wu, Y.; Sun, F.; Liu, Z., Enhancing hydrovoltaic power generation through heat conduction effects. *Nature Communications* **2022,** *13* (1), 1043.
38. Li, L.; Feng, S.; Du, L.; Wang, Y.; Ge, C.; Yang, X.; Wu, Y.; Liu, M.; Wang, S.; Bai, Y., A hydrovoltaic power generation system based on solar thermal conversion. *Nano Energy* **2022,** *99*, 107356.
39. Chen, Y.; He, J.; Ye, C.; Tang, S., Achieving Ultrahigh Voltage Over 100 V and Remarkable Freshwater Harvesting Based on Thermodiffusion Enhanced Hydrovoltaic Generator. *Advanced Energy Materials* **2024**, 2400529.
40. Ren, G.; Hu, Q.; Ye, J.; Hu, A.; Lü, J.; Zhou, S., All-biobased hydrovoltaic-photovoltaic electricity generators for all-weather energy harvesting. *Research* **2022**.
41. Li, X.; Feng, G.; Chen, Y.; Li, J.; Yin, J.; Deng, W.; Guo, W., Hybrid hydrovoltaic electricity generation driven by water evaporation. *Nano Research Energy* **2024,** *3*, e9120110.
42. Xu, T.; Ding, X.; Shao, C.; Song, L.; Lin, T.; Gao, X.; Xue, J.; Zhang, Z.; Qu, L., Electric Power Generation through the Direct Interaction of Pristine Graphene-Oxide with Water Molecules. *Small* **2018,** *14* (14), 1704473.
43. Li, L.; Hao, M.; Yang, X.; Sun, F.; Bai, Y.; Ding, H.; Wang, S.; Zhang, T., Sustainable and flexible hydrovoltaic power generator for wearable sensing electronics. *Nano Energy* **2020,** *72*, 104663.
44. Kumar, R.; Tabrizizadeh, T.; Chaurasia, S.; Liu, G.; Stamplecoskie, K., Hydrovoltaic power generation from multiwalled carbon nanotubes. *Sustainable Energy & Fuels* **2022,** *6* (4), 1141-1147.
45. Kumar, R.; Kay, G.; Beaton, G.; Liu, G.; Stamplecoskie, K., Tuning the functionalization of graphite for hydrovoltaic power generation. *ACS Applied Materials & Interfaces* **2023,** *15* (5), 7511-7517.
46. Shao, B.; Song, Z.; Chen, X.; Wu, Y.; Li, Y.; Song, C.; Yang, F.; Song, T.; Wang, Y.; Lee, S.-T., Bioinspired hierarchical nanofabric electrode for silicon hydrovoltaic device with record power output. *ACS nano* **2021,** *15* (4), 7472-7481.
47. Garemark, J.; Ram, F.; Liu, L.; Sapouna, I.; Cortes Ruiz, M. F.; Larsson, P. T.; Li, Y., Advancing hydrovoltaic energy harvesting from wood through cell wall nanoengineering. *Advanced Functional Materials* **2023,** *33* (4), 2208933.
48. Liu, C.; Wang, S.; Wang, X.; Mao, J.; Chen, Y.; Fang, N. X.; Feng, S.-P., Hydrovoltaic energy harvesting from moisture flow using an ionic polymer–hydrogel–carbon composite. *Energy & Environmental Science* **2022,** *15* (6), 2489-2498.
49. Li, L.; Zheng, Z.; Ge, C.; Wang, Y.; Dai, H.; Li, L.; Wang, S.; Gao, Q.; Liu, M.; Sun, F., A flexible tough hydrovoltaic coating for wearable sensing electronics. *Advanced Materials* **2023,** *35* (40), 2304099.
50. Yoon, S. G.; Yang, Y.; Yoo, J.; Jin, H.; Lee, W. H.; Park, J.; Kim, Y. S., Natural evaporation-driven ionovoltaic electricity generation. *ACS Applied Electronic Materials* **2019,** *1* (9), 1746-1751.
51. Ni, F.; Xiao, P.; Zhang, C.; Zhou, W.; Liu, D.; Kuo, S. W.; Chen, T., Atmospheric hygroscopic ionogels with dynamically stable cooling interfaces enable a durable thermoelectric performance enhancement. *Advanced Materials* **2021,** *33* (49), 2103937.
52. Wu, H.; Zheng, H.; Qin, X.; Jin, Y.; Li, Y.; Yang, S.; Yi, Z.; Gao, S.; Wang, S.; Wang, Z., Drinking-bird-enabled triboelectric hydrovoltaic generator. *Device* **2024,** *2* (5).
53. Huangfu, X.; Guo, Y.; Mugo, S. M.; Zhang, Q., Hydrovoltaic Nanogenerators for Self-Powered Sweat Electrolyte Analysis. *Small* **2023,** *19* (15), 2207134.

54. Liu, J.; Huang, L.; He, W.; Cai, X.; Wang, Y.; Zhou, L.; Yuan, Y., Moisture-enabled hydrovoltaic power generation with milk protein nanofibrils. *Nano Energy* **2022,** *102*, 107709.
55. Qin, Y.; Wang, Y.; Sun, X.; Li, Y.; Xu, H.; Tan, Y.; Li, Y.; Song, T.; Sun, B., Constant Electricity Generation in Nanostructured Silicon by Evaporation-Driven Water Flow. *Angewandte Chemie International Edition* **2020,** *59* (26), 10619-10625.
56. Xue, G.; Xu, Y.; Ding, T.; Li, J.; Yin, J.; Fei, W.; Cao, Y.; Yu, J.; Yuan, L.; Gong, L., Water-evaporation-induced electricity with nanostructured carbon materials. *Nature nanotechnology* **2017,** *12* (4), 317-321.
57. Wang, H.; Sun, Y.; He, T.; Huang, Y.; Cheng, H.; Li, C.; Xie, D.; Yang, P.; Zhang, Y.; Qu, L., Bilayer of polyelectrolyte films for spontaneous power generation in air up to an integrated 1,000 V output. *Nature Nanotechnology* **2021,** *16* (7), 811-819.
58. Huang, Y.; Cheng, H.; Yang, C.; Yao, H.; Li, C.; Qu, L., All-region-applicable, continuous power supply of graphene oxide composite. *Energy & Environmental Science* **2019,** *12* (6), 1848-1856.
59. Cheng, H.; Huang, Y.; Zhao, F.; Yang, C.; Zhang, P.; Jiang, L.; Shi, G.; Qu, L., Spontaneous power source in ambient air of a well-directionally reduced graphene oxide bulk. *Energy & Environmental Science* **2018,** *11* (10), 2839-2845.
60. Huang, Y.; Cheng, H.; Yang, C.; Zhang, P.; Liao, Q.; Yao, H.; Shi, G.; Qu, L., Interface-mediated hygroelectric generator with an output voltage approaching 1.5 volts. *Nature Communications* **2018,** *9* (1), 4166.
61. Liu, X.; Gao, H.; Ward, J. E.; Liu, X.; Yin, B.; Fu, T.; Chen, J.; Lovley, D. R.; Yao, J., Power generation from ambient humidity using protein nanowires. *Nature* **2020,** *578* (7796), 550-554.
62. Li, M.; Zong, L.; Yang, W.; Li, X.; You, J.; Wu, X.; Li, Z.; Li, C., Biological Nanofibrous Generator for Electricity Harvest from Moist Air Flow. *Advanced Functional Materials* **2019,** *29* (32), 1901798.
63. Shen, D.; Xiao, M.; Zou, G.; Liu, L.; Duley, W. W.; Zhou, Y. N., Self-Powered Wearable Electronics Based on Moisture Enabled Electricity Generation. *Advanced Materials* **2018,** *30* (18), 1705925.
64. Lee, S.; Eun, J.; Jeon, S., Facile fabrication of a highly efficient moisture-driven power generator using laser-induced graphitization under ambient conditions. *Nano Energy* **2020,** *68*, 104364.
65. Gao, X.; Xu, T.; Shao, C.; Han, Y.; Lu, B.; Zhang, Z.; Qu, L., Electric power generation using paper materials. *Journal of Materials Chemistry A* **2019,** *7* (36), 20574-20578.
66. Han, Y.; Wang, Y.; Wang, M.; Dong, H.; Nie, Y.; Zhang, S.; He, H., Nanofluid-guided Janus membrane for high-efficiency electricity generation from water evaporation. *Advanced Materials* **2024,** *36* (23), 2312209.
67. Liu, H.; Cui, P.; Zhang, J.; Wang, J.; Ge, Y.; Zhou, Z.; Meng, Y.; Huang, Z.; Yang, K.; Du, Z., Harnessing natural evaporation for electricity generation using MOF-based nanochannels. *Small* **2024,** *20* (36), 2400961.
68. Li, L.; Gao, S.; Hao, M.; Yang, X.; Feng, S.; Li, L.; Wang, S.; Xiong, Z.; Sun, F.; Li, Y., A novel, flexible dual-mode power generator adapted for wide dynamic range of the aqueous salinity. *Nano Energy* **2021,** *85*, 105970.
69. Zhang, J.; Hou, Y.; Lei, L.; Hu, S., Moist-electric generators based on electrospun cellulose acetate nanofiber membranes with tree-like structure. *Journal of Membrane Science* **2022,** *662*, 120962.
70. Shi, X.; Wei, Y.; Tang, B.; Li, Y.; Lv, L.; Lin, S.; Luo, S.; Wang, T.; Tan, S.; Sun, Q., A Kirigami-Driven Stretchable Paper-Based Hydrovoltaic Electricity Generator. *Advanced Functional Materials* **2025**, 2419753.

71. Liu, X.; Ueki, T.; Gao, H.; Woodard, T. L.; Nevin, K. P.; Fu, T.; Fu, S.; Sun, L.; Lovley, D. R.; Yao, J., Microbial biofilms for electricity generation from water evaporation and power to wearables. *Nature Communications* **2022,** *13* (1), 4369.
72. Wang, H.; He, T.; Hao, X.; Huang, Y.; Yao, H.; Liu, F.; Cheng, H.; Qu, L., Moisture adsorption-desorption full cycle power generation. *Nature Communications* **2022,** *13* (1), 2524.
73. Zhao, Q.; Wen, H.; Wu, J.; Wen, X.; Xu, Z.; Duan, J., Galactomannan/graphene oxide/Fe3O4 hydrogel evaporator for solar water evaporation for synergistic photothermal power generation. *Desalination* **2024,** *570*, 117064.
74. Yuan, B.; Yang, L.; Yang, H.; Bai, L.; Wang, W.; Wei, D.; Liang, Y.; Chen, H., Flexible vacancy-mediated MoS2-x nanosheet arrays for solar-driven interfacial water evaporation, photothermal-enhanced photodegradation, and thermoelectric generation. *Energy Conversion and Management* **2022,** *252*, 115070.
75. Fu, Z.; Zhong, D.; Zhou, S.; Zhang, L.; Long, W.; Zhang, J.; Wang, X.; Xu, J.; Qin, J.; Gong, J., Scalable asymmetric fabric evaporator for solar desalination and thermoelectricity generation. *Advanced Science* **2024,** *11* (45), 2406474.
76. Ma, Y.; Hu, Y.; Li, N.; Wang, Y.; Yu, J.; Hu, Z., Integrated Extraction of Electrical Energy and Freshwater from Seawater via Asymmetric Evaporator. *Advanced Functional Materials* **2025**, 2422725.
77. Liu, D.; Wang, D.; Hong, T.; Wang, Z.; Wang, Y.; Qin, Y.; Su, L.; Yang, T.; Gao, X.; Ge, Z., Lattice plainification advances highly effective SnSe crystalline thermoelectrics. *Science* **2023,** *380* (6647), 841-846.
78. Zhao, Y. D.; Jiang, W.; Zhuo, S.; Wu, B.; Luo, P.; Chen, W.; Zheng, M.; Hu, J.; Zhang, K.-Q.; Wang, Z.-S.; Liao, L.-S.; Zhuo, M.-P., Stretchable photothermal membrane of NIR-II charge-transfer cocrystal for wearable solar thermoelectric power generation. *Science Advances* **2023,** *9* (50), eadh8917.
79. Hu, Q.; Ma, Y.; Ren, G.; Zhang, B.; Zhou, S., Water evaporation–induced electricity with Geobacter sulfurreducens biofilms. *Science Advances* **2022,** *8* (15), eabm8047.
